# Nucleoside conjugates of quantum dots for characterization of G protein-coupled receptors: strategies for immobilizing A_2A _adenosine receptor agonists

**DOI:** 10.1186/1477-3155-8-11

**Published:** 2010-05-17

**Authors:** Arijit Das, Gangadhar J Sanjayan, Miklós Kecskés, Lena Yoo, Zhan-Guo Gao, Kenneth A Jacobson

**Affiliations:** 1 Laboratory of Bioorganic Chemistry, National Institute of Diabetes and Digestive and Kidney Diseases, National Institutes of Health, Bethesda, Maryland 20892, USA

## Abstract

**Background:**

Quantum dots (QDs) are crystalline nanoparticles that are compatible with biological systems to provide a chemically and photochemically stable fluorescent label. New ligand probes with fluorescent reporter groups are needed for detection and characterization of G protein-coupled receptors (GPCRs).

**Results:**

Synthetic strategies for coupling the A_2A _adenosine receptor (AR) agonist CGS21680 (2-[4-(2-carboxyethyl)phenylethylamino]-5'-*N*-ethylcarboxamidoadenosine) to functionalized QDs were explored. Conjugates tethered through amide-linked chains and poly(ethyleneglycol) (PEG) displayed low solubility and lacked receptor affinity. The anchor to the dendron was either through two thiol groups of (R)-thioctic acid or through amide formation to a commercial carboxy-derivatized QD. The most effective approach was to use polyamidoamine (PAMAM) D5 dendrons as multivalent spacer groups, grafted on the QD surface through a thioctic acid moiety. In radioligand binding assays, dendron nucleoside conjugate 11 displayed a moderate affinity at the human A_2A_AR (K_iapp _1.02 ± 0.15 μM). The QD conjugate of increased water solubility 13, resulting from the anchoring of this dendron derivative, interacted with the receptor with K_iapp _of 118 ± 54 nM. The fluorescence emission of 13 occurred at 565 nm, and the presence of the pendant nucleoside did not appreciably quench the fluorescence.

**Conclusions:**

This is a feasibility study to demonstrate a means of conjugating to a QD a small molecular pharmacophore of a GPCR that is relatively hydrophobic. Further enhancement of affinity by altering the pharmacophore or the linking structures will be needed to make useful affinity probes.

## Background

Quantum dots (QDs) are crystalline semiconducting nanoparticles that, when properly derivatized, are compatible with biological systems to provide a chemically and photochemically stable fluorescent label [1]. The spectral characteristics are dependent on the particle size, which typically ranges from 2 - 10 nm, resulting in emission wavelengths in the 500 - 800 nm range. QDs have been chemically functionalized, leading to specfic interactions with cellular components for the purposes of biological imaging and therapeutics [2]. For example, antibodies have been covalently coupled to QDs for detection of tumors by confocal microscopy or whole body imaging using a near-infrared label [3-7]. In some cases, small molecular fluorescent prosthetic groups were superior to QDs as a mean of labeling cancer-related receptor sites to follow their regulation [8].

**Figure 1:**
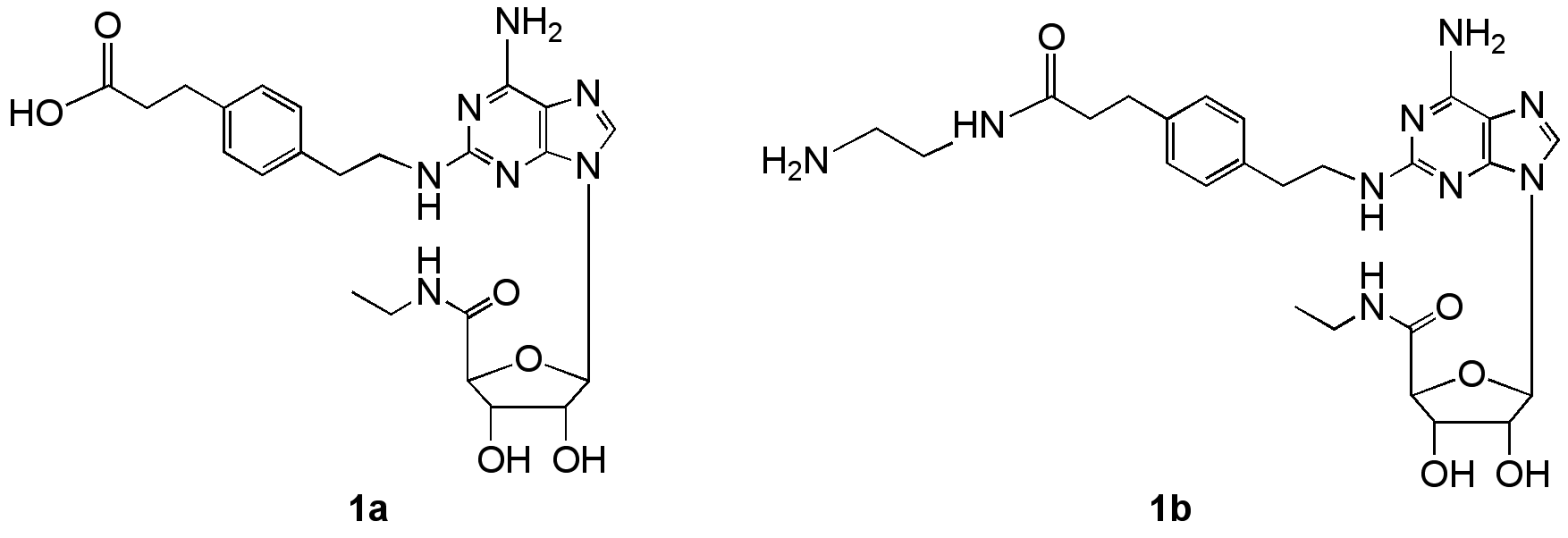
**Structures of the A_2A_AR functionalized agonists congeners used in this study: the carboxylic acid derivative 1a and amine derivative 1b**.

G protein-coupled receptors (GPCRs) are important pharmaceutical targets on the cell surface. We have developed a general approach toward functionalization of small molecular ligands of GPCRs that allow them to be conjugated to carriers, coupled to other pharmacophores, or immoblized on polymers without losing the ability to bind to the receptor with high affinity [9]. In fact, the attachment of functionalized congeners to carriers has resulted in great increases in the potency and selectivity of various GPCR ligands [10-12]. Previously, we have coupled agonists of the antiinflammatory A_2A _adenosine receptor (AR) to polyamidoamine (PAMAM) dendrimers as carriers, with the retention of high affinity and functional potency [10]. Although small-molecule agonists of GPCRs, including ARs [13,14], generally bind within the transmembrane domains, proper functionalization of the ligand makes it possible to overcome the steric limitations of receptor binding. The nucleoside-based agonist CGS21680 (2-[4-(2-carboxyethyl)phenylethylamino]-5'-*N*-ethylcarboxamidoadenosine, 1a, and its ethylenediamine adduct APEC, 1b, Figure 1) [15] were suitable functionalized congeners for this purpose [16].

New ligand probes with fluorescent reporter groups are needed for detection and characterization of GPCRs. Here, we applied QDs to the study of GPCRs in which the native ligand is a small molecule. Previously, peptide ligands and small neurotransmitter-like molecules were coupled to QDs resulting in specific interactions with the target receptors and drug transporters [17,18]. Antibodies to cannabinoid and glutamate receptors were also conjugated to QDs to follow the fate of the receptors [19].

This is a feasibility study to show how a small molecular pharmacophore of a GPCR that is relatively hydrophobic may be conjugated to a QD and still interact with the receptor. We have compared several approaches to the derivatization of CdSe/ZnS QDs to achieve conjugation of active agonists of the A_2A_AR. The problems of limited aqueous solubility of the QD [20-23] and access of the flexible tethered agonist to its transmembrane binding site on the receptor [9] were addressed, resulting in significant AR affinity binding of one QD conjugate. The issue of internal quenching, as observed from dopamine conjugates of QDs [24], has also been explored.

## Results

This study was designed to probe the feasibility of binding QDs to the human A_2A_AR expressed in mammalian cells using covalently tethered nucleoside agonist ligands. Various approaches to the linking chemistry and the nature of the spacer group and solubilizing groups were compared. The QD nucleoside conjugates and their underivatized precursor QDs are shown in Table 1 (2 - 13). Structures of these derivatives are shown schematically in Additional file 1, Table S1.

**Table 1:** *In vitro *pharmacological data for various QDs, dendrons (D5), and their complexes with nucleosides and solubilizing moieties.

Compd.	K_iapp _at hA_2A_AR, μM or *% inhibition*^a^	Solubility
**1a**	0.015	+++

**1b**	0.010	+++

**2a**	NT	-

**2b**	NE^e^	+++

**3**	NE^e^	++

**4**	*< 20%*^e^	+

**5**	*< 20%*^e^	-

**6**	*< 20%*^e^	+

**7**	*< 20%*^e^	+

**8^b^**	*< 20%*^e^	++(72.3 nM in DMSO)^d^

**9**	*< 20%*^e^	++

**10**	*9.8 ± 7.4% *(at 1.0 μM)	+++

**11**	1.02 ± 0.15	+++

**12**	*2.2 ± 1.1% *(at 1.0 μM)	+++

**13**^c^	0.118 ± 0.054	+++ (66.1 μM in DMSO)^d^

^a ^All experiments were done on HEK-293 cells stably expressing the human A_2A_AR. The binding affinity (n = 3-5) and was determined by using agonist radioligands [^3^H]CGS21680. The concentrations of the ligand complexes were measured by the concentration of the macromolecule, not the attached nucleoside. Therefore, binding K_i _values calculated from the IC_50 _using the Cheng-Prusoff equation[37] of large conjugates are expressed as K_iapp _values.

^b ^**8**, MRS5252.

^c ^**13**, MRS5303.

^d ^In order to determine more exactly the solubility of the compounds in two cases we plotted a standard curve graph. We measured the fluorescence intensity of the underivatized QDs (**2a **and **2b**) in DMSO at different concentrations; then, we measured the fluorescence intensity of each conjugate, **8 **and **13**, in DMSO to determine its maximal solubility, based on comparison to the standard curve of the chemical precursor **2a **or **2b**.

^e ^NE, no effect, or less than 20% inhibition at the maximal concentration tested. This concentration was intended to be 1 μM, however in most cases this was not reached due to precipitation.

NT, not tested.

### Synthesis of QD Conjugates of Agonist Functionalized Congeners of the A_2_AR - CGS21680 and APEC

Three approaches to immobilizing functionalized AR agonist ligands to QDs have been used. Nucleoside derivatives, A_2_AR agonists that were prefunctionalized for covalent coupling to carriers were used: the carboxylic acid CGS21680 1a and the primary amine APEC 1b.

**Figure 2:**
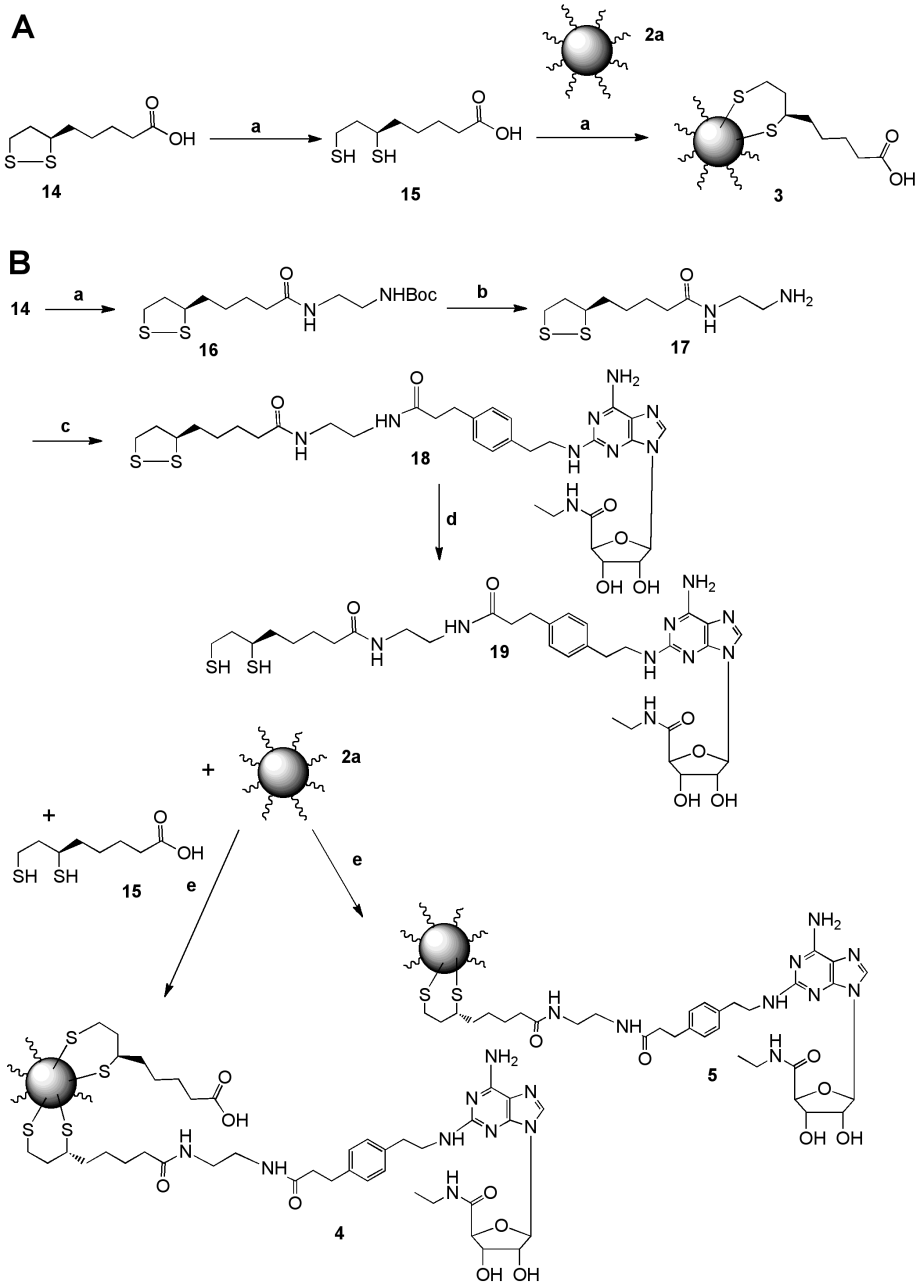
**A. Synthesis of QD conjugate of (R)-thioctic acid 3**. *Reagents and conditions*: (a) NaBH_4_, EtOH, H_2_O; (b) CdS/ZnS QD (**2a**, toluene-soluble), DMSO, EtOH, 60-80°C. B. Synthesis of QD-nucleoside conjugates **4 **and **5 **linked through amide chains that are anchored on the QD surface through the thiol groups of thioctic acid. (a) *N*-Boc-ethylenediamine, DCC, DMAP, DCM; (b) TFA:DCM (1:1); (c) CGS21680 **1a**, DIEA, PyBOP, DMF; (d) Solid phase NaBH_4 _bead, DMF, EtOH, H_2_O; (e) CdS/ZnS (QD) (**2a**, toluene-soluble), DMSO, EtOH, 60-80°C. The number of adenosine moieties attached per QD was approximately 100-180 for conjugate **5 **and 50-110 for conjugate **4**.

**Figure 3:**
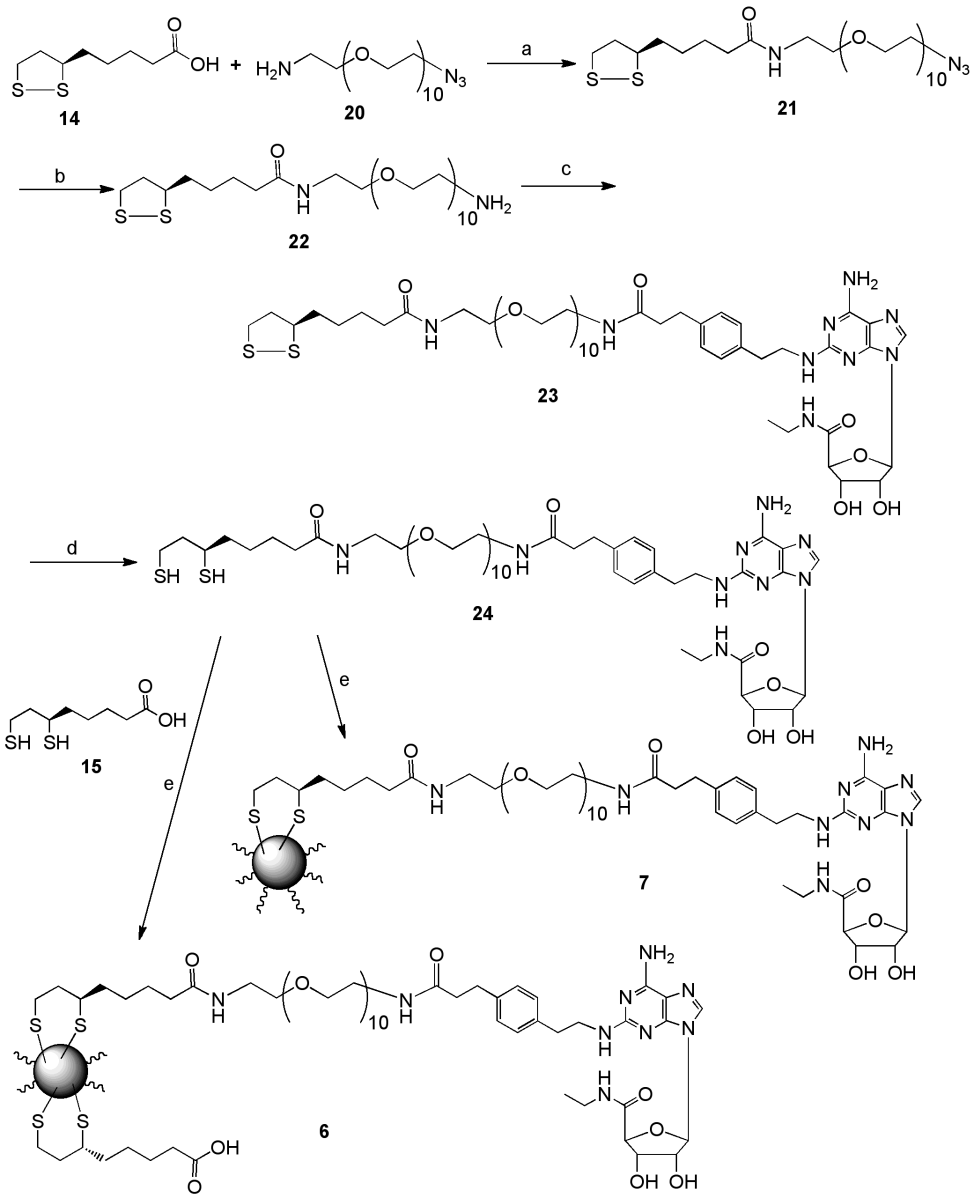
**Synthesis of PEGylated QD conjugates 6 and 7, coupled through a PEG-linked thioctic acid moiety**. *Reagents and conditions*: (a) DCC, DMAP, DCM; (b) PPh_3_, THF, H_2_O; (c) CGS21680 **1a**, DIEA, PyBOP, DMF; (d) Solid-supported BH_4_^+ ^bead, DMF, EtOH, H_2_O; (e) CdS/ZnS QD (toluene-soluble), DMSO, EtOH, 60-80°C. The number of adenosine moieties attached per QD was approximately 100-180 for conjugate **7 **and 50-110 for conjugate **6**.

In Figures 2 and 3, (R)-thioctic acid (TA, -lipoic acid 14, or its reduced dihydro form 15) was used as an anchoring moiety for chains containing a single nucleoside moiety. The route in Figure 2 utilized an exclusively amide-linked chain, and in Figure 3 an intervening poly(ethyleneglycol) (PEG) spacer group of ten units was present within the chain between the nucleoside moiety and the TA anchor. The free thiol groups displaced the native caps (trioctylphosphine/trioctylphosphine oxide) present on the surface of the commercial toluene-soluble QD 2a to form a stable covalent anchor. Thus, two different chain lengths were used in direct conjugation of individual nucleoside units to the hydrophobic QD surface: a short chain containing an ethylenediamine spacer in 4 and 5, and a long chain containing a PEG spacer in 6 and 7. In conjugates 4 and 6, there was an optional cofunctionalization of the QD surface with free TA as a means of increasing compatibility with aqueous medium.

**Figure 4:**
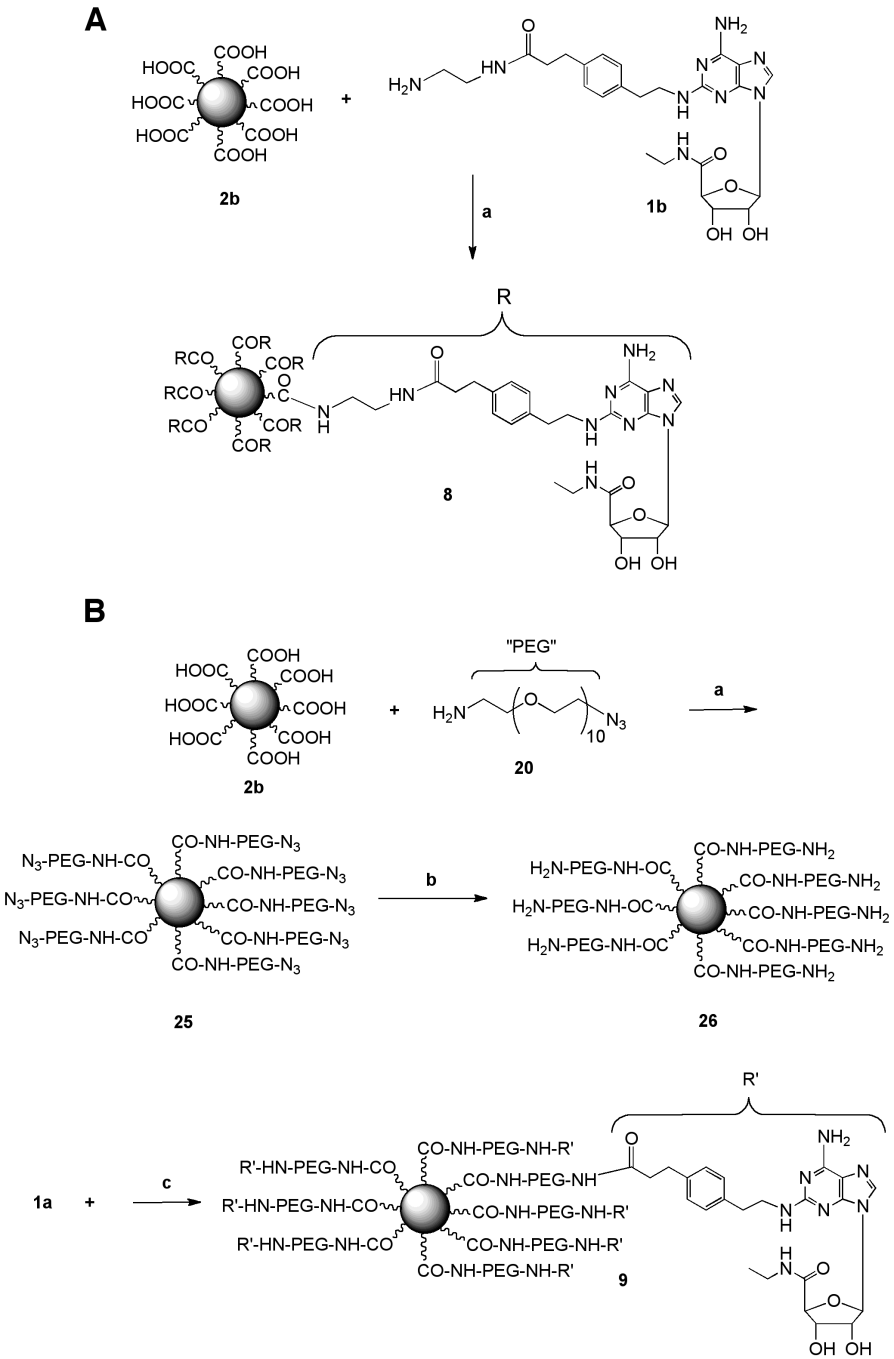
**A. Synthesis of QD conjugate 8 based on a surface-coated carboxylic acid QD 2b**. *Reagents and conditions*: (a) EDC, *N*-hydroxysuccinimide, PBS, DMSO. B. Synthesis of QD conjugate **9 **based on a surface-coated carboxylic acid dendrimer **2b **and coupled through a PEG-linker. *Reagents and conditions*: (a) EDC, *N*-hydroxysuccinimide, PBS, DMSO; (b) PPh_3_, THF, H_2_O; (c) CGS21680 **1a**, DIEA, PyBOP, DMF. The degree of nucleoside substitution of the QDs was estimated to be equal to 50-100 on conjugate **8 **and 30-80 on conjugate **9**.

In Figures 4A and 4B, a commercially coated QD containing a hydrophilic polycarboxylic acid surface was used for immobilizing the nucleoside. The carboxylic coating served both to increase the aqueous solubility of the QD and to be used as a convenient handle for derivatization. The nucleoside was incorporated covalently either as the amine-functionalized congener 1b amide-coupled directly leading to 8 or by the coupling of 1a through a long-chain PEG spacer group of ten units present in 9.

**Figure 5:**
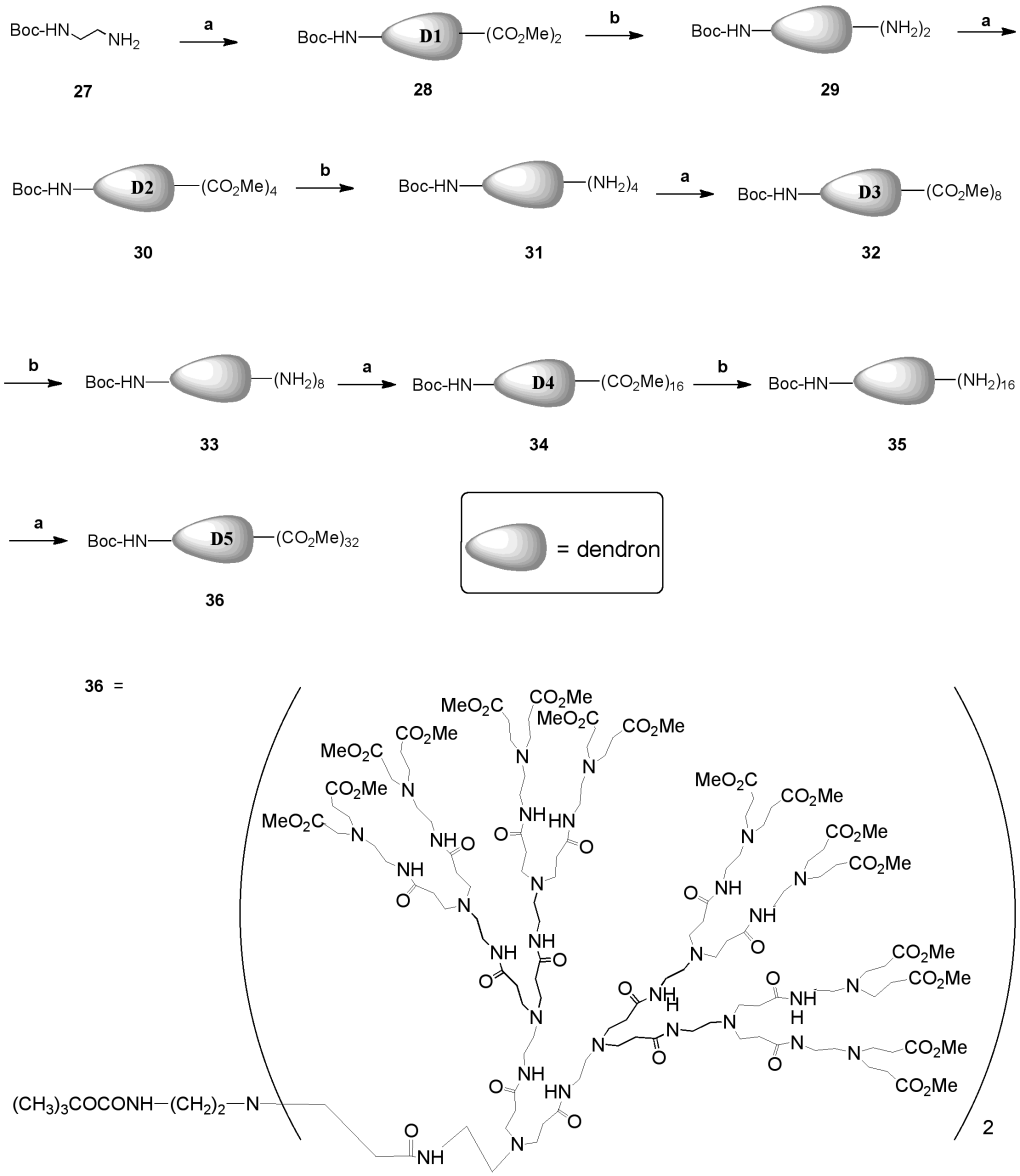
**Synthesis of D5 dendron derivative 36**. *Reagents and conditions*: (a) methyl acrylate (excess), MeOH, 48 h, RT; (b) ethylenediamine (excess), MeOH, 5 d, -10°C.

**Figure 6:**
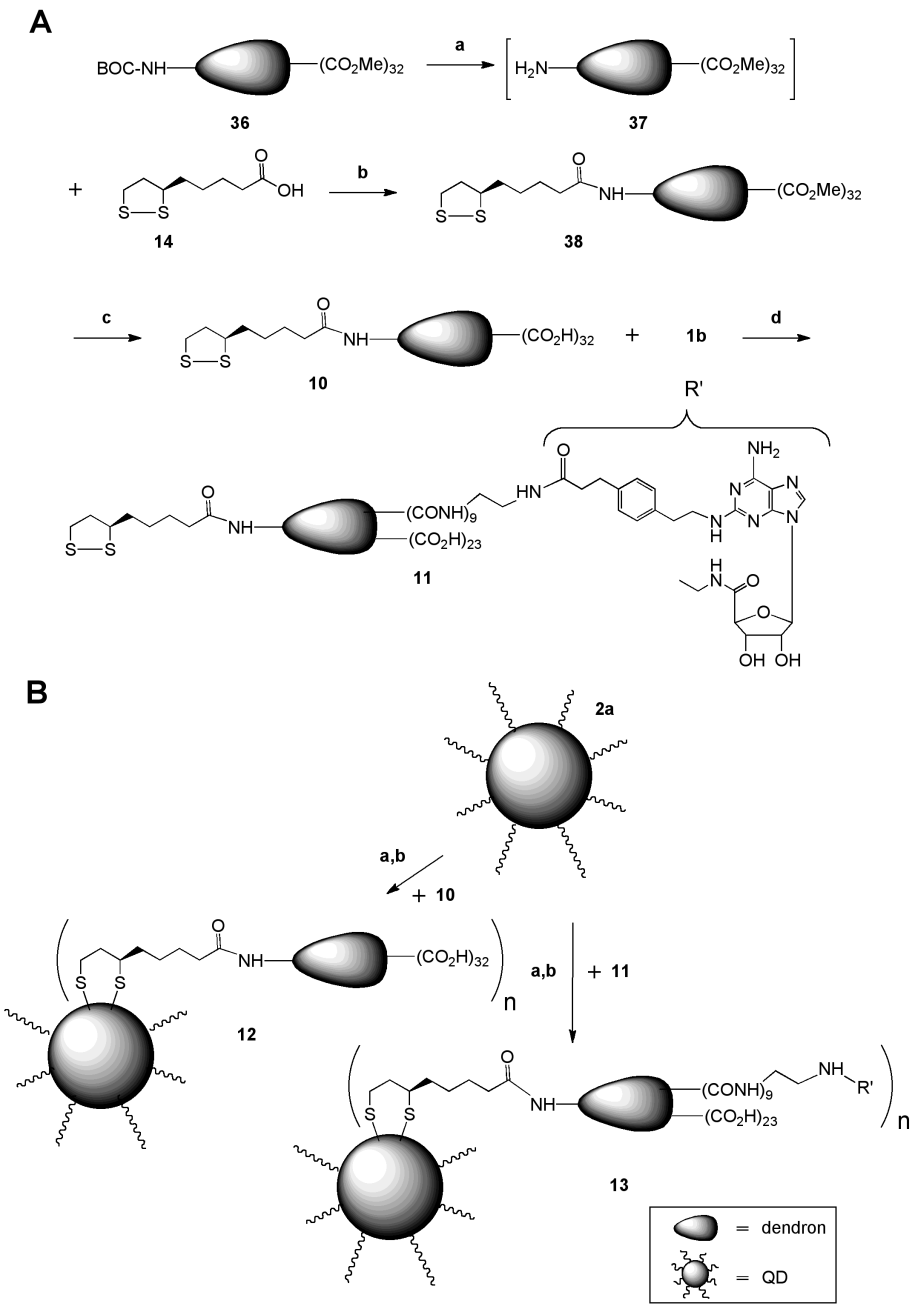
**A. Synthesis of dendron conjugate 11**. B. Synthesis of QD conjugates **12 **and **13. ***Reagents and conditions*: (a) TFA:DCM (1:1); (b) EDC, *N*-hydroxysuccinimide, DMF; (c) LiOH, MeOH, H_2_O (c) APEC (**1b**), DIEA, PyBOP, DMSO; (e) Solid-supported BH_4_^+ ^bead, DMF, EtOH, H_2_O; (f) CdS/ZnS QD (toluene-soluble), DMSO, EtOH, 60-80°C. The degree of dendron substitution of the QDs, variable n, was estimated to be equal to ~100-150 on conjugate **12 **and ~100-150 on conjugate **13 **(see text).

In Figures 5 and 6, we have introduced a PAMAM dendron of generation 5 (D5) as a surface coating and drug-linking moiety to greatly enhance the aqueous solubility of the QD and to increase the nucleoside loading. This dendron is to serve as an intervening "soft" multivalent spacer between the nucleoside and the surface of the QD, which is a "hard" nanoparticle [25,26]. Using a common dendrimer synthesis route shown in Figure 5, we have synthesized an ester form of the dendron 36, which contains a single Boc-protected amine to anchor the dendron onto the QD surface. The maximal number of peripheral groups on each D5 dendron unit (*i.e*. number of esters in 36) was 32. The synthesis was carried out by an iterative method that is standard for the preparation of PAMAM dendrimer derivatives, involving repetitive Michael addition-amidation cycles (Figure 5). Commercially available *N*-Boc-ethylenediamine 27 was first subjected to bis-Michael addition using an excess of methyl acrylate in methanol, affording the Michael adduct (dendron D1) 28 in good yield, which was then subjected to amidation using excess of ethylenediamine in methanol to yield the bis-amine 29. Extension of this repetitive cycle eventually furnished the D5 dendron 36.

Compound 36 was deprotected at a single site with TFA to provide a free amino group, which was coupled condensation to TA using the water-soluble carbodiimide EDC (14) to produce compound 38 (Figure 6) [27]. The peripheral ester groups of compound 38 were saponified with lithium hydroxide to obtain 10, which was coupled with APEC 1b. The product amide, compound 11, contained an estimated 8 - 10 nucleoside moieties per dendron. QD dendron conjugates 12 (control nanocarrier) and 13 (drug-loaded nanocarrier containing the nucleoside-bearing dendron) were prepared from 10 and 11, respectively. In compound 12, we have attached to the QD only the dendron that contains many carboxylic acid groups at its periphery, which are intended to increase the water solubility.

### Pharmacological Characterization of Nucleoside Conjugates of QDs

The affinity of the QD conjugates was examined in a standard radioligand binding assay using [^3^H]1a in membranes of human embryonic kidney (HEK-293) cells expressing the human A_2A_AR (Table 1) [11]. The thiotic-acid anchored derivatives nucleoside derivatives 4-**7 **and the amide-anchored derivative 8 and 9 were inactive or only weakly inhibited binding at the human A_2A_AR at the highest concentration used (1 μM). It is likely that the limited aqueous solubility impaired the binding assay, resulting in precipitation/nondissolution of the nonpolar QD derivatives [28]. For example, a short-chain nucleoside conjugate 8 of the water-soluble QD displayed subthreshold affinity at the human A_2A_AR, with only a small percent of inhibition of radioligand binding. A spacer consisting of a ten-unit PEG chain in 9 did not enhance the ability to measure the affinity at the receptor.

**Figure 7:**
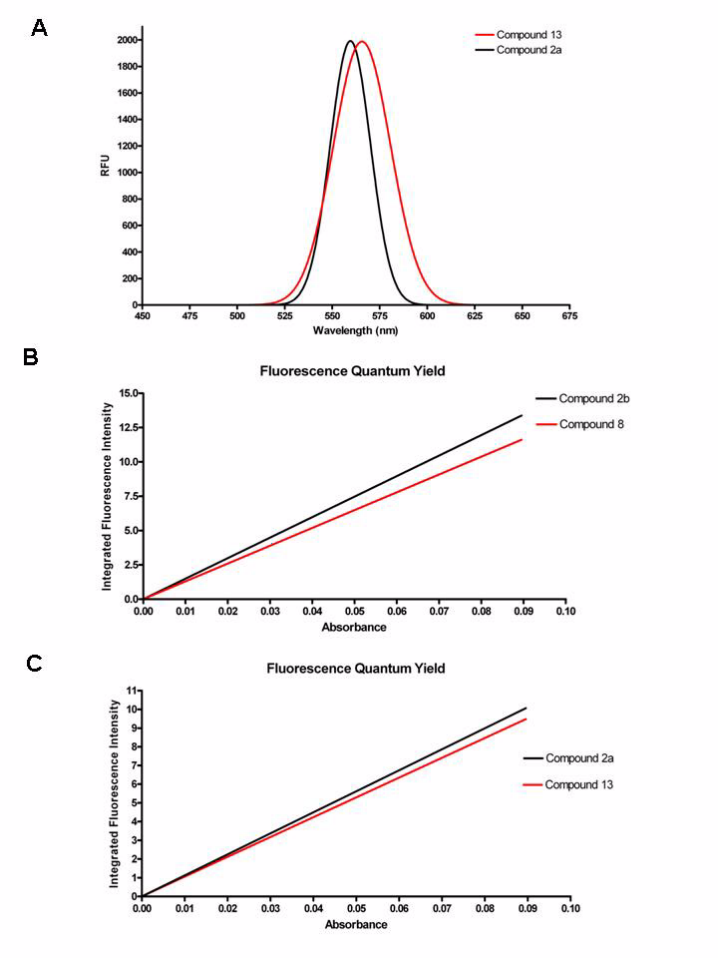
**Fluorescence characteristics of QDs and dendron-linked nucleoside conjugate**. A) Fluorescence emission spectrum of the free QD **2a **and compound **13**. _max _of free QD **2a **= 560 nm; _max _of compound **13 **= 565 nm. B) Linear plots of the free water-soluble QD **2b **and compound **8**. C) Linear plots for the free toluene-soluble QD **2a **and compound **13**. The slope of each line is proportional to the fluorescence quantum yield (Φ_F_) of each sample.

However, compound 13 provided a potent K_i _value (118 ± 54 nM), in comparison to the micromolar K_i _value (1.02 ± 0.15 μM) of the dendron-nucleoside precursor 11. The affinity of compound 13 at the human A_1 _and A_3_ARs was too weak for the determination of K_i _values. The percent displacement of radioligands by 1 μM 13 was 8.6 ± 8.6% and 18.5 ± 1.6%, respectively, at human A_1 _and A_3_ARs in membranes of stably transfected CHO cells. The fluorescence emission of 13 occurred at 565 nm. The fluorescent emission maximum of the free QD was 560 nm, and therefore the fluorescent spectrum did not change significantly (Figure 7A). We measured the fluorescence quantum yield (Φ_F_) of the free QDs in order to determine the fluorescent efficiency of compound 13 and 8. The Φ_F _is the ratio of photons absorbed to photons emitted through fluorescence. We used the comparative method by Williams *et al. *[29], which involves the use of a standard sample with a known Φ_F _value. The Φ_F _of the underivatized QDs is 50% according to the supplier. The compounds 13 and 8 have lower Φ_F _values, but these values are also appropriate for use of these compounds as fluorescent probes (Figures 7B, 7C) and showed that the presence of the pendant nucleoside did not appreciably quench the fluorescence.

## Discussion

We have attached nucleosides that are agonists of the Gs-coupled A_2A_AR to nanocrystalline, inorganic fluorophores (QDs) of great intensity and stability, for the eventual application to receptor imaging and characterization [30]. Although QDs are already used extensively in flow cytometry and imaging based on antibody conjugation, there are few examples of their use with covalently-bound ligands of GPCRs. We have compared various approaches to couple the nucleoside in a manner that retains its ability to interact with the receptor. QDs are "hard" nanoparticles and dendrimers are "soft", using a recently introduced scheme for categorizing nanomaterials [25,26]. Our approach was to enhance both the solubility and the ability of QD derivatives to interact with "soft" biopolymers, such as receptors, by coating the "hard" nanoparticle core with a dendritic "soft" shell. This also facilitated the loading of the drug/ligand onto the surface, by preconjugation to the dendron spacer.

Thus, it was necessary to greatly enhance the water solubility of the QD by changing the surface chemistry. TA groups and PEG chains were previously reported to increase the water solubility of QDs to facilitate their use in biological systems. However, since the presence of functionalized AR agonist reduced the solubility of the QDs even further, those derivatization approaches were inadequate in this study. Coating the surface of 4 and 6 with TA moieties, which were also used to tether the nucleoside, did not create sufficient water solubility to adequately determine the AR binding affinity. Only when D5 dendrons were used as the intervening linkage, was the water solubility sufficient to measure a K_i _value. Also, it was necessary to exhaustively wash the QD derivatives to avoid residual monomers, which would make it appear to be more potent in receptor binding. For example, before measuring the binding of conjugate 8, the QD particles were washed by successive centrifugations. Even after 5 cycles of such washing, there was some residual AR binding present, evidently from the monomer. When more than 5 washing cycles were applied to 8, the percent inhibition of radioligand binding at any QD concentration tested was well below 50%.

**Figure 8:**
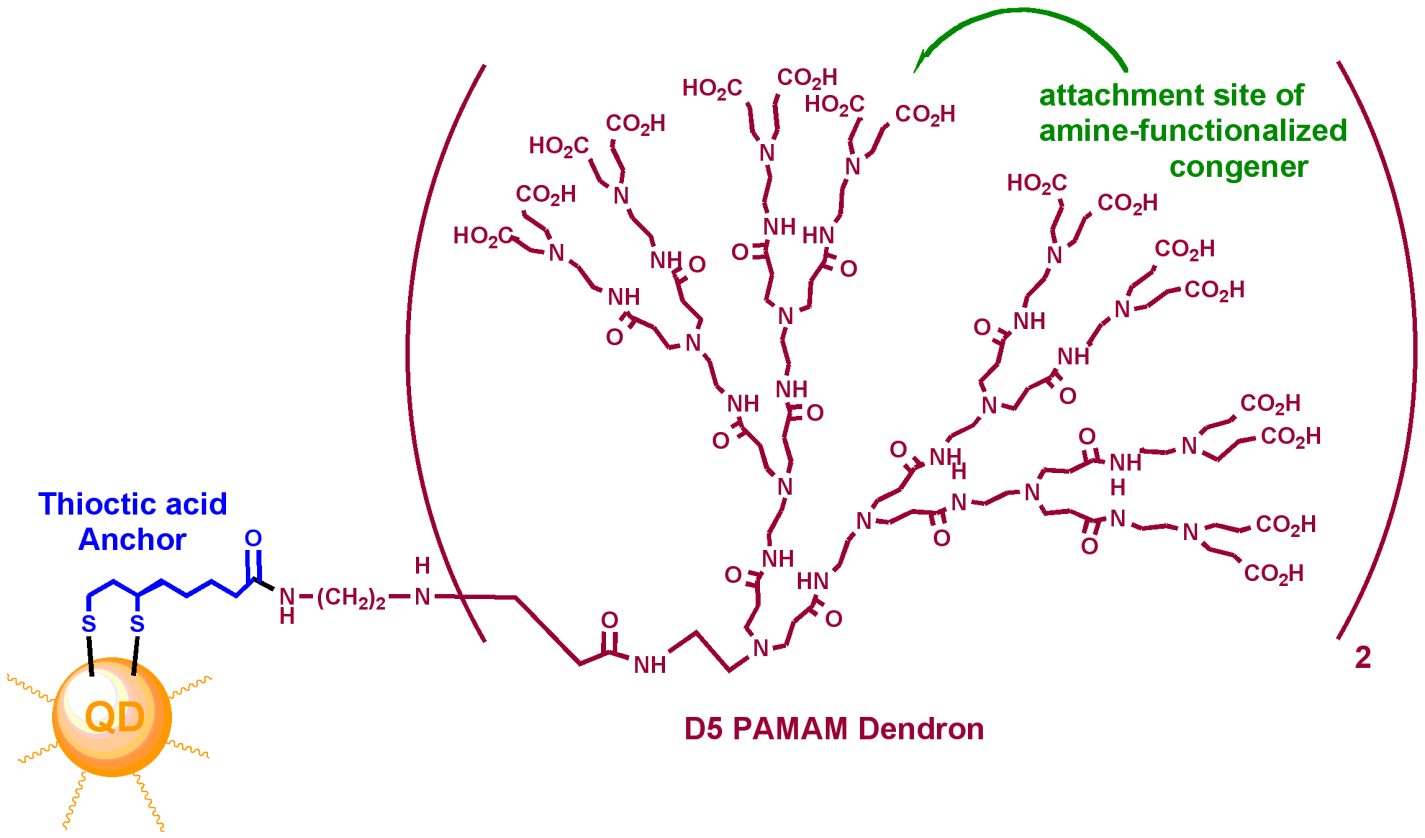
**General design features of QD conjugate 13, which bound with submicromolar affinity to the A_2A_AR**. The QD is represented here as a small sphere, but its diameter is approx. 6 nm, which is several times larger than the size of the appended dendron.

The affinity achieved in the QD conjugate 13 containing the PAMAM dendron linker (Figure 8) was even greater than that of the dendron-nucleoside conjugate 11, suggesting that loss of affinity is not a necessary consequence upon tethering a small molecular GPCR ligand to a QD. Although the conjugates prepared in this study are not of sufficiently high affinity to optimally serve as tracers in receptor binding or histochemical experiments, this is an exploration of the feasibility of this chemical approach for linking small and somewhat hydrophobic GPCR ligands. The intervening dendron not only increases the theoretical stoichiometry of substitution with the ligand, but it also greatly enhances the water solubility. Future structural exploration might identify other QD-bound ligands or nucleoside linkages to provide higher receptor affinity than was observed here. Nevertheless, we have overcome the limitations of physical properties preventing the effective binding of such QD conjugates to a GPCR. Additional studies will determine if nM affinities can be reached using this approach. Also, QDs of different composition, e.g. alloyed CdTeSe/CdS QDs as near infrared optical probes, have been demonstrated to be biocompatible for long-term *in vivo *imaging [31]. The dendrimeric tethering approach for GPCR ligands could potentially be applied to other types of QDs.

## Conclusions

Our long-term objective is to create novel and practical ligand tools needed to characterize GPCRs and their drug interactions. This study is a prototypical example of the design of quantum dot conjugates as fluorescent, multivalent nanocarriers for small molecular ligands, such as adenosine, that bind to and activate GPCRs. These receptors, which are important therapeutic and analytical targets, are soft biopolymers that occur on the surface of cells. The binding sites for molecules like adenosine are buried within the transmembrane cleft of each receptor, which is embedded in a phospholipid bilayer cell membrane. The ability to measure the receptor affinity depended greatly on the type of coating and covalent linkage to the QD. Conjugates tethered as monovalent attachments through amide-linked chains and PEG displayed low solubility and lacked receptor affinity. The most effective approach was to use PAMAM D5 dendrons as multivalent spacer groups, grafted on the QD surface through a TA moiety, which suitably increased water solubility and maintained the ability of the QD conjugate to bind to the GPCR. Thus, in order to effectively bind a hard nanocrystal such as a QD to a receptor for a small molecular ligand, it was necessary to coat the core with a multivalent soft shell, i.e. in our study a dendron linker, which also served as the site for drug tethering. The resulting geometry both enhanced water solubility of the nanoparticle derivative and permitted the nucleoside moiety to penetrate into its binding cleft. Further enhancement of affinity by altering the pharmacophore or the linking structures will be needed to make useful affinity probes.

Certainly, these findings suggest that ligands tethered on dendrimeric spacers attached to QDs could provide a general approach to image GPCRs for small molecular ligands. This method would not be limited to the A_2A_AR, which was explored here as a test case. The GPCRs are important drug targets, and application of QD technology to this receptor superfamily could be very useful in diagnostics, drug screening, and research.

## Methods

### Chemical Synthesis

#### Materials

All reactions were carried out under nitrogen atmosphere using dry solvents. TA, azido-PEG-amine (mol. wt. 526), ethylenediamine-*N*-Boc, DCC, 4-*N, N*-dimethylaminopyridine, trifluoroacetic acid, NaBH_4_, EtOH, triphenylphosphine, dichloromethane, DMF, tetrahydrofuran, PyBOP, diisopropylethylamine, polymer-supported borohydride (on Amberlyst^® ^IRA-400, Macroporous, 20-50 mesh, ~2.5 mmol/g loading, cat. No. 328642-25G) and EDC were purchased from Sigma Aldrich. Solutions of QDs in toluene 2a (CdSe/ZnS, Cat. No. QSO-560-0050, 50 nmol/mL) and in water (CdSe/ZnS Quantum Dots with Carboxylic Acid Surface Groups 2b, Cat. No. QSH-550-20, 8 nmol/mL) were purchased from Ocean NanoTech (Springdale, AR), and CGS21680 was purchased from Tocris Chemicals (Ellisville, MO). APEC 1b bistrifluoroacetic acid was provided by NIMH Chemical Synthesis and Drug Supply Program.

#### Chromatography and spectroscopy

High performance liquid chromatography (HPLC) purification was performed using an Agilent 1100 Series HPLC (Santa Clara, CA) equipped with a Phenomenex Luna 5 μ C18(2) 100A analytical column (250 × 10 mm; Torrance, CA). Peaks were detected by UV absorption using a diode array detector. IR spectra of the QDs conjugates Additional file 2 were recorded using a PerkinElmer Spectrum One FT-IR spectrometer (applied as a DMSO solution). UV/visible spectra of representative compounds Additional file 3 were measured using a SpectraMax M5 Multi-Mode Microplate reader (Molecular Devices, Sunnyvale, CA). ^1^H NMR spectra Additional file 4 were recorded with a Varian Gemini 300 spectrometer. High-resolution electron spray ionization mass spectra (ESI MS, Additional file 5) were measured on a Water LCT premier mass spectrometer at the Mass Spectrometry Facility, NIDDK, NIH. Standard fluoresent curves Additional file 6 were measured using a SpectraMax M5 Microplate reader.

#### Chemical synthesis - (R)-Thioctic acid-ethylenediamine-N-Boc (16) and (R) Thioctic acid-PEG40000-azide (21)

The synthesis followed a published procedure [32]. (R)-Thioctic acid 14 (206 mg, 1 mmol), *N*-Boc-ethylenediamine (160 mg, 1 mmol) for 16 or NH_2_-PEG-N_3 _20 (526 mg, 1 mmol) for compound 21, and DMAP (36 mg, 0.3 mmol) were stirred with 20 mL DCM and cooled to 0°C. DCC (206 mg, 1 mmol) was added to the reaction mixture under a nitrogen atmosphere, and the mixture was stirred at that temperature for 2.5 h and then warmed to room temperature, where stirring continued overnight. The reaction mixture was filtered through a short Celite column using ethyl acetate, and the filtrate was evaporated leaving the crude products 16 and 21, respectively. The crude product was then purified by silica gel column chromatography using DCM:MeOH (10:1, by volume).

Compound 16 (247.7 mg, 71%) was obtained as a yellowish liquid. The NMR spectrum was consistent with the assigned structure. ^1^H NMR (300 MHz, CD_3_OD) δ 6.3 (m, 1 H), 5.01 (m, 1 H), 3.55 (m, 1 H), 3.38 (m, 2 H), 3.31 (m, 2 H), 3.11 (m, 2 H), 2.41 (m, 1 H), 2.18 (t, *J *= 7.5 Hz, 2 H), 1.82 (m, 1 H), 1.62 (m, 4 H), 1.51 (m, 11H). m/z (M ^+^ESI MS) found: 349.1624; calc: 349.1620.

Compound 21 (486 mg, 68%) was obtained as a yellowish gummy solid. ^1^H NMR (CD_3_OD) 6.43 (brs, 1 H), 3.63 (m, peg H), 3.51 (t, *J *= 5.1, 2 H), 3.46 (m, 1 H), 3.40 (t, *J *= 5.1, 2 H), 3.09 (m, 2 H), 2.46 (m, 1 H), 2.21 (t, *J *= 7.4 Hz, 2 H), 1.93 (m, 1 H), 1.72 (m, 4 H), 1.51 (m, 2H). m/z (M ^+^ESI MS) found: 715.3658; calc: 715.3622.

#### (R)-Thioctic acid-ethylenediamine (17)

Compound 16 (348 mg, 1 mmol) was dissolved in 20 mL of 1:1 DCM and TFA (99%), and the mixture was stirred at room temperature for 1 h. The solvent was evaporated under vacuum, and the crude product was successively treated with DCM and evaporated, several times, to yield a reasonably pure compound 17, which was used without purification for the next step. Compound 17 (231.5 mg, 93%) was obtained as a gummy solid. ^1^H NMR (300 MHz, D_2_O) δ 3.51 (m, 1 H), 3.42 (m, 2 H), 3.16 (m, 2 H), 2.93 (m, 2 H), 2.41 (m, 1 H), 2.19 (t, *J *= 7.7 Hz, 2 H), 1.87 (m, 1 H), 1.64 (m, 4 H), 1.51 (m, 2H). m/z (M ^+^ESI MS) found: 249.1106; calc: 249.1095.

#### (R)-Thioctic acid-amino-PEG-amine (22)

The synthesis followed a published procedure [32]. Amino-PEG-azide 21 (714 mg, 1 mmol) (17) and triphenylphosphine (524 mg, 2 equiv., 2 mmol) were stirred with 20 mL of THF at room temperature under a nitrogen atmosphere for 2 h. Water (1 ml, 0.05 mol) was added to the mixture, and the reaction mixture was stirred for 72 h under a nitrogen atmosphere. The solvent was evaporated, and the crude product was purified by silica gel column chromatography using first 10:1 DCM:MeOH and then 100:20:1 DCM:MeOH:Et_3_N as eluent. Compound 22 (572 mg, 83%) was obtained as a yellowish liquid. ^1^H NMR (300 MHz, CDCl_3_) δ 6.54 (brs, 1 H), 3.67 (m, peg H), 3.58 (m, 2 H), 3.49 (m, 1 H), 3.11 (m, 2 H), 2.87 (m, 2 H), 2.76 (brs, 2 H), 2.47 (m, 1 H), 2.16 (m, 2 H), 1.85 (m, 1 H), 1.68 (m, 4 H), 1.48 (m, 2H). m/z (M ^+^ESI MS) found: 689.9310; calc: 689.9318.

#### (R)-Thioctic acid-ethylenediamine-CGS21680 (18) and (R)-Thioctic acid-amino-PEG-amine-CGS21680 (23)

TA-Ethylenediamine compound 17 (for compound 18) (13 mg, 0.05 mmol) or TA-Peg-NH_2 _compound 22 (for compound 23) (35 mg, 0.05 mmol), and CGS21680 (1a, 11 mg, 0.0198 mmol) as a hydrochloride salt were dissolved in 1 mL DMF. Then DIEA (9 μL, 0.05 mmol) was added to the reaction mixture and stirred for 10 min. PyBOP (11 mg, 0.02 mmol) was added to the solution, and the mixture was stirred for 18 h at room temperature. The solvent was removed in vacuum, and the crude product was dissolved in a minimum volume (200 μL) of methanol. An excess volume (5-7 mL) of dry diethyl ether was added, and the mixture was left overnight at 4°C, leading to the precipitation of the product. The ether supernatant was then removed using a Pasteur pipette, and the remaining solid was dried in vacuum to get the pure products 18 and 23.

#### (R)-Thioctic acid-ethylenediamine-CGS21680 (18)

Compound 18 (23 mg, 65%) was obtained as a gummy yellowish solid. ^1^H NMR (300 MHz, DMSO) δ ^1^H NMR (300 MHz, DMSO) δ 8.1, 8.03 (s (each), 2H), 7. 84, 7. 78 (d (each), J = 6.8 Hz, 4 H), 7.1 (br s, 2 H), 5.93 (d, J = 6.3 Hz, 1 H), 5.42 (m, 2 H), 4.46 (m, 1 H), 4.43 (m, 2 H), 3.52 (m, 1 H), 3.38 (m, 6 H), 3.18 (m, 2 H), 2.91 (m, 6 H), 2.45 (m, 1 H), 2.31 (m, 2 H), 2.14 (m, 2 H), 1.83 (m, 1 H), 1.68 (m, 4 H), 1.51 (m, 2 H), 0.91 (t, J = 6.8 Hz, 3H). m/z (M ^+^ESI MS) found: 730.3159; calc: 730.3169.

#### (R)-Thioctic acid-amino-PEG-amino-CGS21680 (23)

Compound 23 (36 mg, 63%) was obtained as a gummy yellowish solid. ^1^H NMR (300 MHz, D_2_O) δ ^1^H NMR (300 MHz, DMSO) δ 8.07 (br s 2 H), 7.85, 7. 78 (d (each), J = 6.8 Hz, 4 H), 6.92 (m, 2 H), 5.95 (d, J = 6.4 Hz, 1 H), 5.41 (m, 2 H), 4. 89 (m, 1 H), 4. 45 (s, 1 H), 4.41 (s, 1 H), 3. 68 (m, peg H), 3.51 (m, 2 H), 3.45 (m, 1 H), 3.32 (m, 6 H), 2.93 (m, 6 H), 2.51 (m, 2 H), 2.24 (t, J = 5.4 Hz, 2 H), 2.15 (m, 2 H), 1.91 (m, 1 H), 1.65 (m, 4 H), 1.43 (m, 2 H), 0.98 (t, J = 6.8, 3H). m/z (M ^+^ESI MS) found: 1170.5853; calc:1170.5790.

#### Synthesis of QD complexes 3, 4, 5, 6, and 7

For the synthesis of conjugates 3, 4, and 7, which were derivatives of the toluene-soluble QDs, we have used free thiol derivatives 15, 19, and 24, respectively. For the preparation of QD conjugates 5 and 6, we have used compounds 15 and 19 or compounds 15 and 24, respectively (each being a 1:1 mixture). Compound 15 was synthesized using the previously reported procedure [32].

Compounds 19 and 24 were also prepared using the similar procedure described earlier [32]. However in this case, we used a solid phase reaction using polymer-supported borohydride beads for the reduction. A solution of compound 18 or 23 (1 equiv) in DMF (1 mL), EtOH (300 μL), and H_2_O (200 μL) was stirred for 10 min at 0°C. Afterwards, the solid-supported borohydride (3.5 equiv mmol, 0.8 gm borohydride resin) was added to the solution, and the reaction mixture was gradually warmed to room temperature. The stirring was continued for 20 h under nitrogen atmosphere. Then the reaction mixture was filtered, and the solvent was evaporated using a rotary evaporator. The presence of SH group in compounds 19 and 24 was confirmed using Ellman's reagent [33]. Since compounds 19 and 24 easily oxidize to the corresponding cyclic compounds 18 and 23, and also because the Ellman test is very sensitive, we used compounds 19 and 24 immediately without purification for the preparation of QD conjugates. 330 μL (16.5 nmol) of a solution of CdSe/ZnS QD 2a in toluene was delivered to a screw cap 1.5 mL plastic Falcon tube, and 300 μL of EtOH was added to the solution. Then, the tube was centrifuged for 30 min at 14,000 rpm, and the supernatant solution was discarded by pipette, and again the QD particles were resuspended using 200 μL of DMSO and EtOH each. After that (200-400 μL) 1000-fold excess compound of 15, 19, or 24 for preparation of 3, 5, or 7 and a 1:1 mixture (200-400 μL, 500-fold excess of each compound) of 15 and 19 or 15 and 24 was added to the solution for the preparation of 4 or 6. The mixture was then heated up to 60-80°C while stirring for 6 h. After homogenization, the mixture was then centrifuged for 30 min at 14000 rpm. The supernatants were discarded, and the pellet was again resuspended in DMSO. The mixture was then heated gently with agitation to maximize the solubility. After cooling to room temperature, the concentration was determined using UV measurement. The number of ligands attached to each QD particle was determined from the UV measurement of the supernatant solutions, which was subtracted from the total amount of ligand used in the reaction. We have assumed no loss of the QD throughout the reaction and centrifugation process. The number of adenosine moieties attached per QD was approximately 100-180 for conjugates 5 and 7 and 50-110 for conjugates 4 and 6. The presence of all the functionality, including TA and CGS21680, was determined by IR spectroscopy of the QD conjugates.

IR spectrum for free QD solution in toluene, cm^-1^: 3027, 1604, 1495, 1459.

IR spectrum for QD conjugate 3 in DMSO, cm^-1^: 3026, 1604, 1498, 1381.

IR spectrum for QD conjugate 4 in DMSO, cm^-1^: 3425, 1666, 1436, 1407, 1311, 1074, 952.

IR spectrum for QD conjugate 5 in DMSO, cm^-1^: 3406, 1661, 1437, 1407, 1314, 1071, 951.

IR spectrum for QD conjugate 6 in DMSO, cm^-1^: 3430, 2996, 2913, 1671, 1436, 1407, 1310, 1071, 952, 930.

IR spectrum for QD conjugate 7 in DMSO, cm^-1^: 3423, 2996, 2913, 1671, 1436, 1407, 1310, 1071, 952, 930.

#### Water-soluble short chain QD-CGS21680 complex (8)

Water-soluble carboxyl terminated QD 2b (50 μL, 0.4 nmole, Ocean Nanotech, cat no: QSH-550-20) and PBS buffer (pH 7.4, 100 μL) was delivered to a 1.5 ml screw cap Falcon tube. EDC (0.03 mg, 400-fold), and NHS (0.18 mg, 400-fold) were added to the solution. The reaction mixture was stirred for 1 h at room temperature. Then, a solution of APEC 1b bistrifluoroacetate salt (1.2 mg, 400-fold, in 200 μL DMSO) was added to the reaction mixture, and the solution was stirred overnight at room temperature. The QD conjugate 8 was purified by centrifugation for 30 min at 14000 rpm. The supernatant was discarded, and the QD solution was resuspended in DMSO (200 μL) followed by centrifugation to remove excess unreacted APEC bistrifluoroacetic acid salt 1b. After discarding the supernatant solution, the QD particles were resuspended in water (40 μL, to a final concentration of 0.01 mM). The number of APEC moieties attached to each QD was determined from UV measurement as described before assuming no loss of the product during the reaction and purification process. Number of APEC moieties attached per QD in compound 8 was approximately 100-150. The presence of APEC in QD conjugate 8 was determined by IR spectroscopy, which showed differences from the free QD 2.

IR spectrum for free QD 2b in water, cm^-1^: 3337, 2105, 1637. IR spectrum for QD conjugate 8 in water, cm^-1^: 3480, 2925, 1637, 1467, 1353, 1103, 1022.

#### Water-soluble long chain QD-PEG-CGS21680 complex (9)

Water-soluble QD 2b (Ocean Nanotech, QSH-550-20, 50 μL, 0.4 nmole), PBS buffer (pH 7.4, 100 μL), EDC (30 μg, 400-fold), and NHS (18 μg, 400-fold) was delivered to a 1.5 mL screw cap Falcon tube. The reaction mixture was stirred for 1 h at room temperature and then NH_2_-PEG-N_3 _(84 μg, 400-fold, in 100 μL DMSO) was added to the reaction mixture. The mixture was stirred overnight at room temperature and then purified by centrifugation using the same procedure as described above (twice using 200 μL DMSO). After discarding the supernatant solution, the QD conjugate 26 was resuspended in 100 μL of water. The presence of an azide group was confirmed using IR spectroscopy.

IR spectra for QD conjugate 26 in water, cm^-1^: 3410, 2873, 2106, 1648, 1437, 1349, 1095, 1071, 951.

Then, the terminal azide of the QD conjugate 25 was reduced using a known procedure [34]. The QD conjugate 25 (0.8 nmol, 100 μL in water) was delivered to a 1.5 mL of screw cap Falcon tube and mixed with THF and triphenylphosphine (117 μg, 448 nmol in 200 μL of THF). The reaction mixture was stirred for 2 h at room temperature under nitrogen. After that 100 μL of water was added to the reaction mixture and the mixture was stirred for 3 days at room temperature under nitrogen atmosphere. The QD conjugate 26 was purified using similar centrifugation procedure (3 times, 30 min each at 14000 rpm, using initially a 1:1 ratio of water:THF; followed by pure THF; and finally pure water, 200 μL each time to remove free triphenyl phosphine). The pure QD conjugate 26 was resuspended in water (100 μL), and the presence of amine and absence of azide was determined using IR.

IR spectrum for QD conjugate 26 in water, cm^-1^: 3380, 1648, 1437, 1407, 1317, 1071, 950.

The final QD conjugate 9was prepared through the coupling of amine-terminated QD derivative 26 and CGS 21680. A mixture of amine-terminated QD derivative 26 (0.4 nmole), CGS 21680 (22 μg, 100 equiv, 40 nmol), DMF (1 mL), and DIEA (11 μg, 85 nmol) was stirred for 10 min. Then PyBOP (42 μg, 80 nmol) was added to the reaction mixture, and the mixture was stirred for 20 h at room temperature. The QD conjugate 9formed was purified using a similar centrifugation procedure consisting of 3 cycles of 30 min each at 14000 rpm, using DMSO (200 μL) each time to remove free CGS21680 and other monomeric derivatives. The pure QD conjugate 9 was resuspended in 40 μL of water, and the loading of CGS21680 per QD was determined using the same procedure (UV measurement) as determined in case of conjugate 8. The number of attached nucleoside moieties per QD in compound 9 was approximately 30-90. The presence of CGS21680 was confirmed by IR spectroscopy.

IR spectrum for QD conjugate 9 in water, cm^-1^: 3441, 2996, 1661, 1436, 1407, 1310, 1075, 952, 930.

#### Synthesis of Dendron (36)

##### Dendron bis-ester 28

A solution of *N*-Boc-ethylenediamine (0.8 g, 4.99 mmol) 27 dissolved in methanol (3 mL) was slowly added to an ice-cold stirred solution of methyl acrylate (2 mL, 22.2 mmol) dissolved in methanol (2 mL). After the addition, the reaction mixture was stirred at room temperature for 48 h. The reaction mixture was stripped of solvent and excess methyl acrylate under vacuum, and the crude product was purified by column chromatography (5% ethylacetate-pet.ether) to afford the dendron 28 as a viscous, colourless liquid (1.2 g, 72%). ^1^H NMR (600 MHz, DMSO-d_6_) δ 5.10 (br.s, 1H, CONH), 3.55 (s, 6H, CO_2_Me ), 3.05 (m, 2 H), 2.65 (m, 4 H), 2.40 (m, 2 H), 2.35 (m, 4 H), 1.35 (s, 9H, C(CH_3_)_3_). m/z (TOF ES MS^+^) found: 333.2025 (100%, M+1), 334.2163 (M+2), 355.2039 (M+Na); calc: 333.2026 (M+1).

##### Dendron bis-amine 29

A solution of dendron bis-ester 28 (1.1 g, 3.30 mmol) dissolved in methanol (3 mL) was slowly added to an ice-cold stirred solution of ethylenediamine (5 mL, 74.7 mmol) dissolved in methanol (2 mL). After the addition was completed, the reaction mixture was stored at ~ -10°C for five days. The reaction mixture was stripped of solvent and excess ethylenediamine under vacuum, and the crude product was repeatedly co-evaporated (6-8 times) with a mixture of toluene-methanol (9:1, v/v), until ethylenediamine could be judged to be absent by ^1^H NMR. The residue was dried under vacuum (12 h) to afford the dendron bis-amine 29 as a viscous, colourless liquid (0.96 g, 80%). ^1^H NMR (600 MHz, DMSO-d_6_) δ 7. 85 (br.s, 2H, CONH), 6. 56 (br.s, 1 H, Boc-NH), 3.05 (m, 4 H), 2.96 (m, 2 H), 2.68 (m, 4 H), 2.55 (m, 4 H), 2.40 (m, 2 H), 2.16 (m, 4 H), 1.35 (s, 9H, C(CH_3_)_3_). m/z (TOF ES MS^+^) found: 389.2889 (100%, M+1), 390.3030 (M+2), 392.3079 (M+4), 411.2811 (M+Na); calc: 389.2876 (M+1).

##### Dendron tetra-ester 30

Following the similar procedure for the synthesis of dendron bis-ester 28, as described earlier, 30 was obtained as a viscous, colourless liquid (1.32 g, 88%). ^1^H NMR (600 MHz, DMSO-d_6_) δ 7. 70 (br.s, 2H, CONH), 6. 50 (br.s, 1H, Boc-NH), 3.48 (s, 12H, CO_2_Me), 3.15 (m, 3 H), 3.05 (m, 4 H), 2.95 (m, 2 H), 2.65 (m, 11 H), 2.60 (m, 12 H), 2.15 (m, 4 H), 1.35 (s, 9H, C(CH_3_)_3_). m/z (TOF ES MS^+^) found: 733.50 (100%, M+1), 734.50 (M+2), 735.50 (M+3), 755.50 (M+Na); calc: 733.43 (M+1).

##### Dendron tetra-amine 31

Following the similar procedure for the synthesis of dendron bis-amine 29, as described earlier, 31 was obtained as a viscous, colourless liquid (1.18 g, 93%). ^1^H NMR (600 MHz, DMSO-d_6_) δ 7. 80 (br.s, 6H, CONH), 6.51 (br.s, 1H, Boc-NH), 3.18 (m, 4 H), 3.02 (m, 8 H), 2.95 (m, 2 H), 2.65 (m, 12 H), 2.55 (m, 8 H), 2.42 (m, 6 H), 2.18 (m, 12 H), 1.35 (s, 9H, C(CH_3_)_3_). m/z (TOF ES MS^+^) found: 845.60 (M+1), 846.60 (M+2), 867.60 (M+Na, 100%); calc: 845.60 (M+1).

##### Dendron octa-ester 32

Following the similar procedure for the synthesis of dendron bis-ester 28, as described earlier, 32 was obtained as a viscous, colourless liquid (1.40 g, 77%). ^1^H NMR (600 MHz, DMSO-d_6_) δ 7. 75 (m, 6H, CONH), 6.45 (br.s, 1H, Boc-NH), 3.60 (s, 24 H, CO_2_Me), 3.15 (m, 12 H), 2.90 (m, 2 H), 2.70 (m, 28 H), 2.40 (m, 30 H), 2.18 (m, 12 H), 1.35 (s, 9 H, C(CH_3_)_3_). m/z (TOF ES MS^+^) found: 1533.90 (M+1), 1555.90 (M+Na, 100%), 1557.9 (M+Na+2); calc: 1532.89 (M).

##### Dendron octa-amine 33

Following the similar procedure for the synthesis of dendron bis-amine 29, as described earlier, 33 was obtained as a viscous, colourless liquid (1.386 g, 96%). ^1^H NMR (600 MHz, DMSO-d_6_) δ 7. 85 (m, 14 H, CONH), 6.45 (br.s, 1 H, Boc-NH), 3.10 (m, 34 H), 2.62 (m, 28 H), 2.58 (m, 14 H), 2.40 (m, 14 H), 2.18 (m, 26 H), 1.38 (s, 9 H, C(CH_3_)_3_). m/z (TOF ES MS^+^) found: 1757.90 (M), 1758.90 (M+1), 1759.90 (M+2), 1779.80 (M+Na-1, 100%), 1780.80 (M+Na), 1781.80 (M+Na+1), 1782.90 (M+Na+2); calc: 1757.23 (M).

##### Dendron 16-ester 34

Following the similar procedure for the synthesis of dendron bis-ester 28, as described earlier, 34 was obtained as a viscous, colourless liquid (1.90 g, 86%). ^1^H NMR (600 MHz, DMSO-d_6_) δ 7. 60 - 7.80 (m, 14 H, CONH), 6.50 (br.s, 1 H, Boc-NH), 3.60 (s, 48 H, CO_2_Me), 3.20 (m, 33 H), 3.15 (m, 25 H), 2.90 (m, 2 H), 2.65 (m, 48 H), 2.40 (m, 48 H), 2.18 (m, 24 H), 1.38 (s, 9 H, C(CH_3_)_3_). m/z (TOF ES MS^+^) found: 3133.80 (M), 3134.80 (M+1), 3135.80 (M+2), 3136.80 (M+3), 3156.80 (M+Na), 3157.80 (M+Na+1); calc: 3133.82 (M).

##### Dendron 16-amine 35

Following the similar procedure for the synthesis of dendron bis-amine 29, as described earlier, 35 was obtained as a viscous, colourless liquid (1.80 g, 92%). ^1^H NMR (600 MHz, DMSO-d_6_) δ 7.85 (m, 30 H, CONH), 6.45 (br.s, 1 H, Boc-NH), 3.15 (m, 76 H), 2.65 (m, 56 H), 2.55 (m, 28 H), 2.45 (m, 28 H), 2.10 (m, 56 H), 1.38 (s, 9 H, C(CH_3_)_3_). m/z (TOF ES MS^+^) found: 3582.50 (M), 3583.50 (M+1), 3584.50 (M+2, 100%), 3585.50 (M+3), 3586.50 (M+4), 3587.50 (M+5); calc: 3582.50 (M).

##### Dendron 32-ester 36

Following the similar procedure for the synthesis of dendron bis-ester 28, as described earlier, 36 was obtained as a viscous, colourless liquid (1.50 g, 54%). ^1^H NMR (600 MHz, DMSO-d_6_) δ 7.82 (m, 14 H, CONH), 7.60 (m, 16 H, CONH), 6.45 (br.s, 1 H, Boc-NH), 3.60 (s, 96 H, CO_2_Me), 3.20 (m, 52 H), 3.15 (m, 54 H), 2.65 (m, 106 H), 2.40 (m, 108 H), 2.18 (m, 52 H), 1.35 (s, 9 H, C(CH_3_)_3_). m/z (TOF ES MS^+^) found: 6340.0 (M+1, 100%), 6362.0 (M+Na); calc: 6339.44 (M).

#### (R)-Thioctic acid-Dendron-conjugate 37

*N*-Boc Dendron 36 (6.2 mg, 1 μmol) was subjected to deprotection of the t-Boc group by exposing to DCM:TFA (1:1, v/v) for 1h at room temperature, followed by evaporation under reduced pressure to furnish the amine as a gummy solid 37 (5.8 mg, 93%).^ 1^H NMR (400 MHz, D_2_O) δ 3.59 (m, 96 H), 3.41 (m, 54 H), 3.39 (m, 52 H), 3.21 (m, 106 H), 2.81 (m, 108 H), 2.63 (m, 52 H). m/z (M ^+^ESI MS) found:6221; calc:6239. The crude material 37 was carried over to the next step without further purification. (R)-Thioctic acid (20 mg, 0.1 mmol) 14, N-hydroxysuccinimide (23 mg, 0.2 mmol), and EDC (57 mg. 0.3 mmol) were dissolved in DMF (5 mL) in a 25 mL round bottom flask. The reaction mixture was stirred for 2 hr, and the crude dendron amine from the previous step (499 mg, 0.08 mmol) and triethylamine (14.6 μL, 0.2 mmol) were added. The reaction mixture was stirred overnight and purified by extensive dialysis over water (4 times, 12 h). The conjugate 38 (516.3 mg, 83%) was obtained as a gummy solid. ^1^H NMR (400 MHz, D_2_O) δ 3.65 (m, 96 H), 3.55 (m, 1 H), 3.41 (m, 54 H), 3.31 (m, 52 H), 3.21 (m, 2 H), 3.01 (m, 106 H), 2.74 (m, 109 H), 2.58 (m, 54 H), 2.1 (m, 1 H), 1.73 (m, 4 H), 1.51 (m, 2H). m/z (M ^+^ESI MS) found: 6452 (with sodium), calc: 6427. IR: (DMSO) cm^-1^: 2940, 1781, 1735, 1712, 1669, 1389, 1177, 1128, 1070.

#### (R)-Thioctic acid-Carboxylic acid terminal dendron conjugate (10)

Compound 38 (311 mg, 0.05 mmol) was dissolved in 10 mL of water and methanol (1:2). LiOH (134 mg, 64 equiv., 3 mmol) was added to the solution and the mixture was stirred overnight. The product was purified by extensive dialysis over water (4 times, 12 h each). Compound 10 (219.5 mg, 71%) was obtained as a gummy solid. ^1^H NMR (400 MHz, D_2_O) δ δ 3.42 (m, 55H (52 from dendrimer, from TA - SCH, SCH_2_)), 3.31 (m, 52 H (from dendrimer)), 3.1 (m, 106 H (from dendrimer)), 2.85 (m, 2H (from dendrimer)), 2.61 (m, 109 H (108 from dendrimer, from TA - CH)), 2.14 (m, 54 H (52 from dendrimer, from TA - CH_2_)), 2.01 (m, 1 H, from TA - CH)), 1.31 (m, 4 H, from TA - 2 CH_2_), 1.21 (m, 2 H, from TA - CH_2_)). m/z (M ^+^ESI MS) found: 6185, calc: 5978. IR: (DMSO) cm^-1^: 3303, 1638, 1409.

#### (R)-Thioctic acid-Dendron-APEC conjugate (11)

Compound **10 **(30 mg, 5 μmol) and APEC 1b bistrifluoroacetic acid (58 mg, 15 equiv. 75 μmol) were dissolved in DMSO (2 mL). DIEA (40 δL, 230 δmol) and PyBOP (465 δL, 33.3 mM solution in DMSO, 15.5 δmol) were added to the mixture. The reaction mixture was stirred for 36 h and purified by dialysis against water and DMF (1:1, water:DMF, 12 h, 4 times). Compound 11 (37 mg, 66%) was obtained as a yellowish gummy solid. Compound 11 The loading of APEC was calculated from the mass spectrum indicating that 10.2 APEC 1b moieties were present per dendron molecule. ^1^H NMR (400 MHz, DMSO-*d*_6_) δ 12.0 (m, 21.8 H (21.4 from free COOH )), 8.08 (m, 82 H (31 from dendrimer NH, 5 from APEC NH)), 8.01 (m, 20.4 H (2 from APEC)), 7.31 (d, J = 7.8 Hz, 20.4 H (2 from aromatic, APEC)), 7.1 (d, J = 7.8 Hz, 20.4 H (2 from aromatic, APEC)), 6.65 (m, 10.2 H (1 from APEC)), 5.24 (m, 20.4 H (2 from APEC), 4.11 (m, 30.6 H (3 from APEC)), 3.61 (m, 95.8 H (52 from dendrimer, 4 from APEC, 3 from TA)), 3.03 (m, 115.2 H (52 from dendrimer, 6 from APEC, 2 from TA)), 2.58 (m, 107 H (106 from dendrimer, 1 from TA)), 2.1 (m, 151.8 H (108 from dendrimer, 4 from APEC, 3 from TA)), 1.56 (m, 56 H (52 dendrimer, 4 TA)), 0.98 (m, 32.6 H (3 from APEC, 2 from TA)). m/z (M ^+^ESI MS) found: 11342, calc: 11312.

#### QD-Carboxylic acid terminal conjugate and QD-Dendron-APEC conjugates (12) and (13)

Compound 10 (3.1 mg, 0.5 δmol) was dissolved in ethanol (0.5 mL) and water (0.2 mL), while compound 11 (5.6 mg, 0.5 δmol) was dissolved in DMF. Solid-suppoprted borohydride (3.5 equiv. 1.75 δmol) was added to the mixture and the mixture was stirred for 24 h under nitrogen atmosphere. The reaction mixture was filtered and evaporated using a rotary evaporator. The presence of SH was confirmed using Ellman's reagent [33]. Since the free SH group oxidized rapidly to its cyclic compounds 10 and 11 and also because the Ellman test is very sensitive, we have used the product directly without purification for the preparation of QD conjugate.

300 μL (16.5 nM) of a solution of CdSe/ZnS QDs in toluene and 300 μL of EtOH were delivered to a screw cap 1.5 mL Falcon tube, and centrifuged for 30 min at 14000 rpm. The supernatant solution was discarded by pipette. Compound 10 (10 mg, 100-fold excess) in 1:1 DMSO and water (300 δL) or a 20-fold excess (4 mg) of compound 11 in DMSO (300 δL) was added to the QD solution. The mixture was then heated to 60-80°C while stirring for 12 h. The mixture was then centrifuged for 30 min (in 14000 rpm). The supernatant was then discarded and the pellet resuspended in water (300 δL) for compound 12 or in DMSO (300 δL) for compound 13. The washing cycle was done 5 times to assure the complete removal of any unbound dendron. After the fifth wash, the QD solution was resuspended in water (300 δL) or DMSO (300 δL), respectively. The mixture was then heated to maximize the solubility. The concentration was determined by fluorescence measurement. We have assumed no loss of the QD throughout the reaction and centrifugation process. The presence of key functional groups in DHLA (CO and OH), CGS21680 (OH, and NH_2_) was determined by IR spectra of the QD conjugate.

IR spectra for QD conjugate 12 in water, cm^-1^: 3337, 1637, 1010, 950.

IR spectra for QD conjugate 13 in DMSO, cm^-1^: 3419, 3001, 2916, 1658, 1436, 1406, 1313, 1080, 951, 899.

#### Cell Culture and Membrane Preparation

CHO (Chinese hamster ovary) cells stably expressing the recombinant human A_1 _and A_3_ARs and HEK-293 (human embryonic kidney) cells stably expressing the recombinant human A_2A_AR were cultured in Dulbecco's modified Eagle medium (DMEM) and F12 (1:1) supplemented with 10% fetal bovine serum, 100 units/mL penicillin, 100 δg/mL streptomycin, and 2 δmol/mL glutamine. After harvesting, cells were homogenized and suspended. Cells were then centrifuged at 500 *g *for 10 min, and the pellet was resuspended in 50 mM Tris-HCl buffer (pH 7.5) containing 10 mM MgCl_2_. The suspension was homogenized and was then recentrifuged at 20 000 *g *for 20 min at 4°C. The resultant pellets were resuspended in Tris buffer, incubated with adenosine deaminase for 30 min at 37°C, and the suspension was stored at -80°C until the binding experiments. The protein concentration was measured using the BCA Protein Assay Kit from Pierce [35].

#### Radioligand Membrane Binding Studies

Radioligand binding assays were performed on three subtypes of ARs, following the procedure described previously [36]. For the A_2A_AR, membranes (20 g/tube) from HEK-293 cells stably expressing the receptor were incubated with [^3^H]CGS21680 (15 nM) at 25°C for 60 min in 50 mM Tris-HCl buffer (pH 7.4, 10 mM MgCl_2_) and increasing concentrations of the test ligands in a total assay volume of 200 L. Nonspecific binding was determined using 10 δM of 5'-*N*-ethylcarboxamidoadenosine (NECA). Each tube in the binding assay contained 100 μL of membrane suspension (20 μg of protein), 50 μL of agonist radioligand, and 50 μL of increasing concentrations of the test ligands in Tris-HCl buffer (50 mM, pH 7.5) containing 10 mM MgCl_2_. The concentration of the QD-ligand complexes was measured as the concentration of the QD, not the nucleoside ligand. Therefore, all K_i _values are measured as apparent inhibition constant (K_iapp_) values. Nonspecific binding was determined using 5'-*N*-ethylcarboxamidoadenosine at a final concentration of 10 μM diluted in buffer. The mixtures were incubated at 25°C for 60 min. Binding reactions were terminated by filtration through Whatman GF/B filters under a reduced pressure using a MT-24 cell harvester (Brandell, Gaithersburg, MD). Filters were washed three times with 5 mL of 50 mM ice-cold Tris-HCl buffer (pH 7.5). All of the filters were washed 3 times with Tris-HCl, pH 7.5. Filters were placed in scintillation vials containing 5 mL of Hydrofluor scintillation buffer and counted using a Perkin Elmer Liquid Scintillation Analyzer. The K_i _values were determined using GraphPad Prism for all assays.

### Fluorescence measurements

For the determination of the fluorescent emission spectrum and the quantum yield, we used a SpectraMax M5 Microplate Reader. In case of the Φ_F _we diluted four different concentrations of the free QDs and compounds 13 and 8 and recorded the absorbance and fluorescence spectrum using a 450 nm excitation wavelength, respectively. After the measurement we calculated the integrated fluorescence intensity using Prism 4.0 software (GraphPAD, San Diego, CA) from the corrected fluorescence spectrum. Finally, we plotted a graph of integrated fluorescence intensity *vs *absorbance. The gradient of the resulting straight line was proportional of the quantum yield of the sample.

### Statistical Analysis

Binding and functional parameters were calculated using Prism 4.0 software (GraphPAD, San Diego, CA). IC_50 _values obtained from competition curves were converted to *K*_i _values using the Cheng-Prusoff equation [37]. Data were expressed as the mean standard error. Statistical analysis was performed using Analysis of Variance (ANOVA) with post hoc test or Student's test where appropriate, and *P *values less than 0.05 were considered significant.

The abbreviations used are: APEC - 2-[*p*-[2-(2-aminoethyl)aminocarbonyl-ethyl]phenylethylamino]-5 -*N*-ethylcarboxamidoadenosine; AR - adenosine receptor; CHAPS - 3-[(3-cholamidopropyl)dimethylammonio]-1-propanesulfonate hydrate; CHO - Chinese hamster ovary; DCM - dichloromethane; DMEM - Dulbecco's Modified Eagle Media; DMF - *N, N*-dimethylformamide; DMSO - dimethyl sulfoxide; EDC - *N*-(3-dimethylaminopropyl)-*N *-ethylcarbodiimide; EDTA - ethylenediaminetetraacetic acid; ERK - extracellular signal-regulated kinase; ESI - electrospray ionization; GPCR - G protein-coupled receptor; [^3^H]CGS21680 - 2-[*p*-(2-carboxyethyl)phenylethylamino]-5 -*N*-ethylcarboxamidoadenosine; HEK - human embryonic kidney; HEPES - 4-(2-hydroxyethyl)-1-piperazineethanesulfonic acid; MALDI-TOF - matrix assisted laser desorption/ionization time-of-flight; MES - 2-(*N*-morpholino)ethanesulfonic acid; MS - mass spectrometry; NMR - nuclear magnetic resonance; PAMAM - poly(amidoamine); PyBOP - benzotriazol-1-yl-oxytripyrrolidinophosphonium hexafluorophosphate.

## Competing interests

The authors declare that they have no competing interests.

## Authors' contributions

AD and GS did the chemical synthesis, experimental design, and manuscript preparation. ZGG and LY did the pharmacological assays and helped with experimental design. KAJ did experimental design and manuscript preparation. All authors read and approved the final manuscript.

## Supplementary Material

Additional file 1 **Additional Table S1**. Data identical to Table 1 except showing chemical structures schematically Click here for file

Additional file 2 **IR spectra of representative compounds**. Compounds **1a**, **2a**, **2b**, **3a**, **4**, **6**, **10**, and **13** Click here for file

Additional file 3 **UV spectra of representative compounds**. Compounds **7 **and **8** Click here for file

Additional file 4 **^1^H NMR spectra of representative compounds**. Compounds **11**, **32**, **35**, and **36** Click here for file

Additional file 5 **Mass spectra of representative compounds**. Compounds **10**, **23**, **32**, **33**, **35**, **36**, and **37** Click here for file

Additional file 6 **Standard fluoresent curves of representative compounds**. Compounds **2a **and **2b** Click here for file
