# Supplementary material for: Nucleoside conjugates of quantum dots for characterization of G protein-coupled receptors: strategies for immobilizing A2A adenosine receptor agonists
Source: J Nanobiotechnology. 2010 May 17;8:11. doi: 10.1186/1477-3155-8-11 (PMC2883535; doi:10.1186/1477-3155-8-11)
Supplement: Additional file 1 — Additional Table S1. Data identical to Table 1 except showing chemical structures schematically [file 1477-3155-8-11-S1.DOCX]

Title: Additional Table S1

Description: Data identical to Table 1 except showing chemical structures schematically

**Table S1**. *In vitro* pharmacological data for various QDs, dendrons (D5), and their complexes with nucleosides and solubilizing moieties.

R′ = PEG = -(CH_2_)_2_-(O CH_2_CH_2_)_10_-

| Compd. | Name/composition | K_iapp_ at hA_2A_AR, µM  or *% inhibition*^a^ | Solubility |
| --- | --- | --- | --- |
| **1a** | CGS21680 (R′-OH) | 0.015 | +++ |
| **1b** | APEC (R′-NH-(CH_2_)_2_-NH_2_) | 0.010 | +++ |
| **2a** |  (toluene-soluble) | NT | - |
| **2b** |  (water-soluble) | NE^e^ | +++ |
| **3** |  | NE^e^ | ++ |
| **4** |  | *<20%*^e^ | + |
| **5** |  | *<20%*^e^ | - |
| **6** |  | *<20%*^e^ | + |
| **7** |  | *<20%*^e^ | + |
| **8^b^** |  | *<20%*^e^ | ++  (72.3 nM in DMSO)^d^ |
| **9** |  | *<20%*^e^ | ++ |
| **10** |  | *9.8±7.4%*  (at 1.0 µM) | +++ |
| **11** |  | 1.02±0.15 | +++ |
| **12** |  | *2.2±1.1%*  (at 1.0 µM) | +++ |
| **13**^c^ |  | 0.118±0.054 | +++  (66.1 µM in DMSO)^d^ |

^a^ All experiments were done on HEK-293 cells stably expressing the human A_2A_AR. The binding affinity (n = 3-5) and was determined by using agonist radioligands [^3^H]CGS21680. The concentrations of the ligand complexes were measured by the concentration of the macromolecule, not the attached nucleoside. Therefore, binding K_i_ values calculated from the IC_50_ using the Cheng-Prusoff equation [29] of large conjugates are expressed as K_iapp_ values.

^b^ **8**, MRS5252.

^c^ **13**, MRS5303.

^d^ In order to determine more exactly the solubility of the compounds in two cases we plotted a standard curve graph. We measured the fluorescence intensity of the underivatized QDs (**2a** and **2b**) in DMSO at different concentrations; then, we measured the fluorescence intensity of each conjugate, **8** and **13**, in DMSO to determine its maximal solubility, based on comparison to the standard curve of the chemical precursor **2a** or **2b**.

^e^ NE, no effect, or less than 20% inhibition at the maximal concentration tested. This concentration was intended to be 1 µM, however in most cases this was not reached due to precipitation.

NT, not tested.
