# Supplementary material for: Nucleoside conjugates of quantum dots for characterization of G protein-coupled receptors: strategies for immobilizing A2A adenosine receptor agonists
Source: J Nanobiotechnology. 2010 May 17;8:11. doi: 10.1186/1477-3155-8-11 (PMC2883535; doi:10.1186/1477-3155-8-11)
Supplement: Additional file 4 — 1H NMR spectra of representative compounds. Compounds 11, 32, 35, and 36 [file 1477-3155-8-11-S4.DOC]

**Additional information for:**

**Nucleoside conjugates of quantum dots for characterization of G protein-coupled receptors: strategies for immobilizing A_2A_ adenosine receptor agonists**

Arijit Das, Gangadhar Sanjayan, Miklos Kecskes, Lena Yoo, Zhan-Guo Gao, and Kenneth A. Jacobson^*^

Additional file 4

Title: ^1^H NMR spectra of representative compounds

Description: Compounds **11**, **32**, **35**, and **36**

**
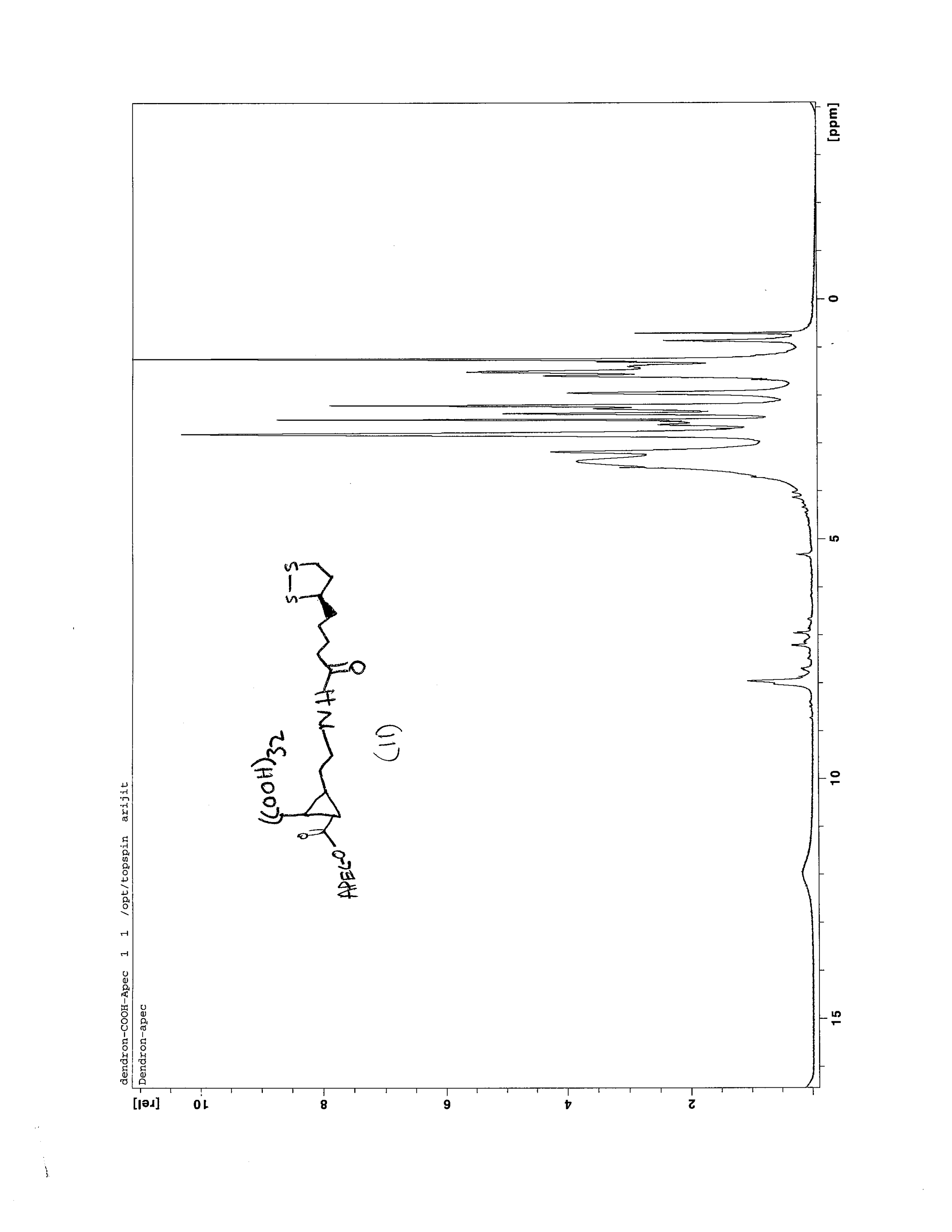
**

**
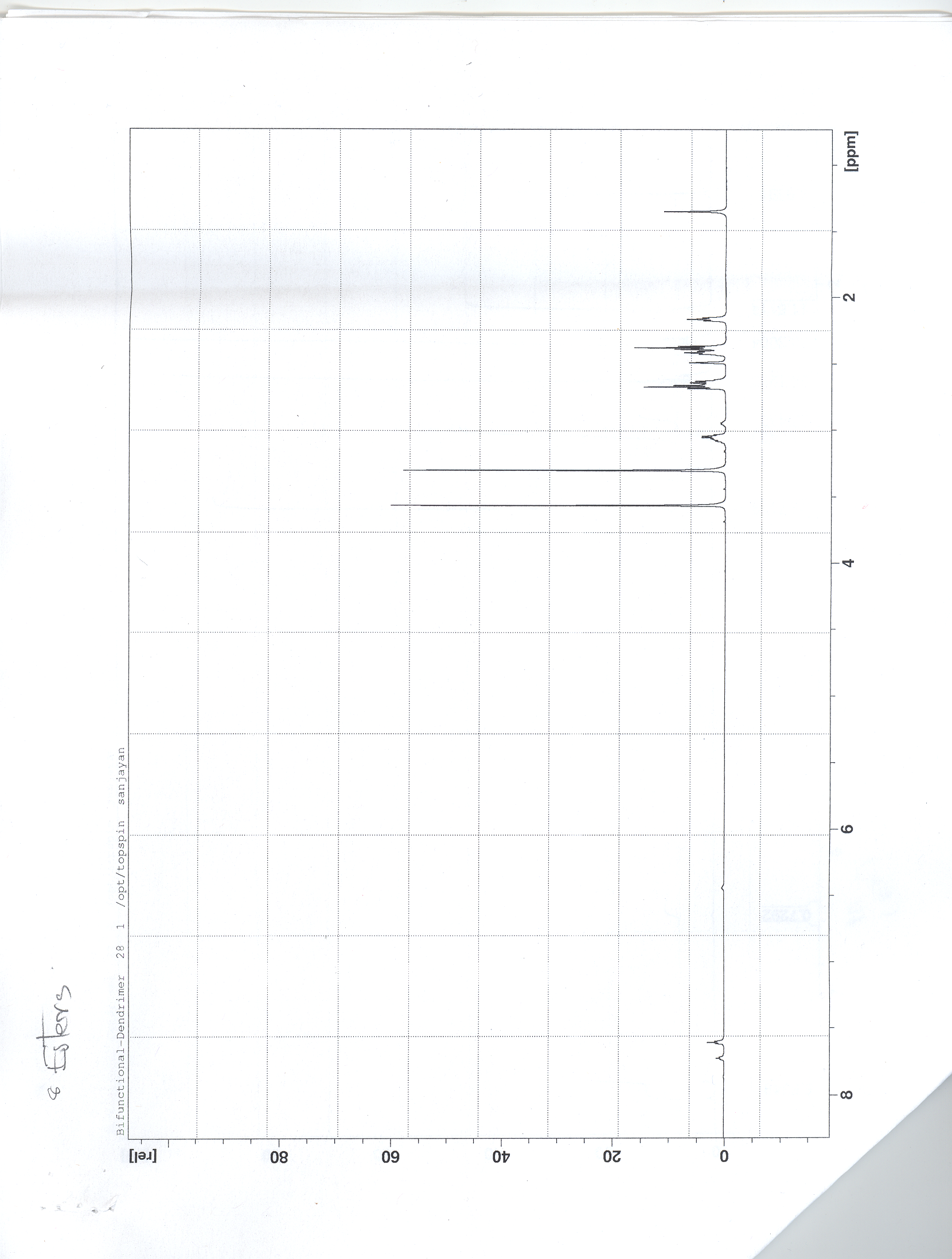

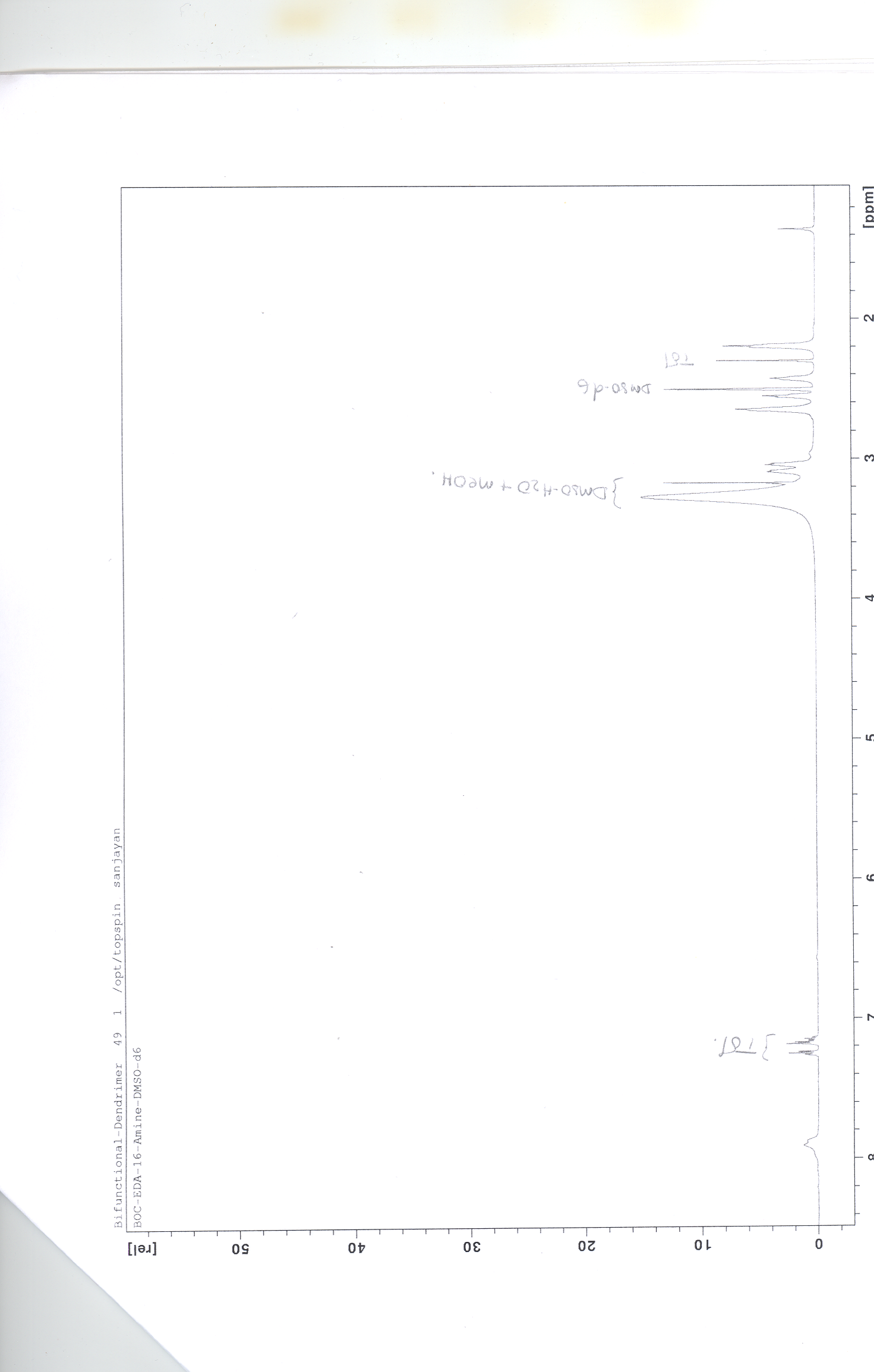
**

MeOH

**
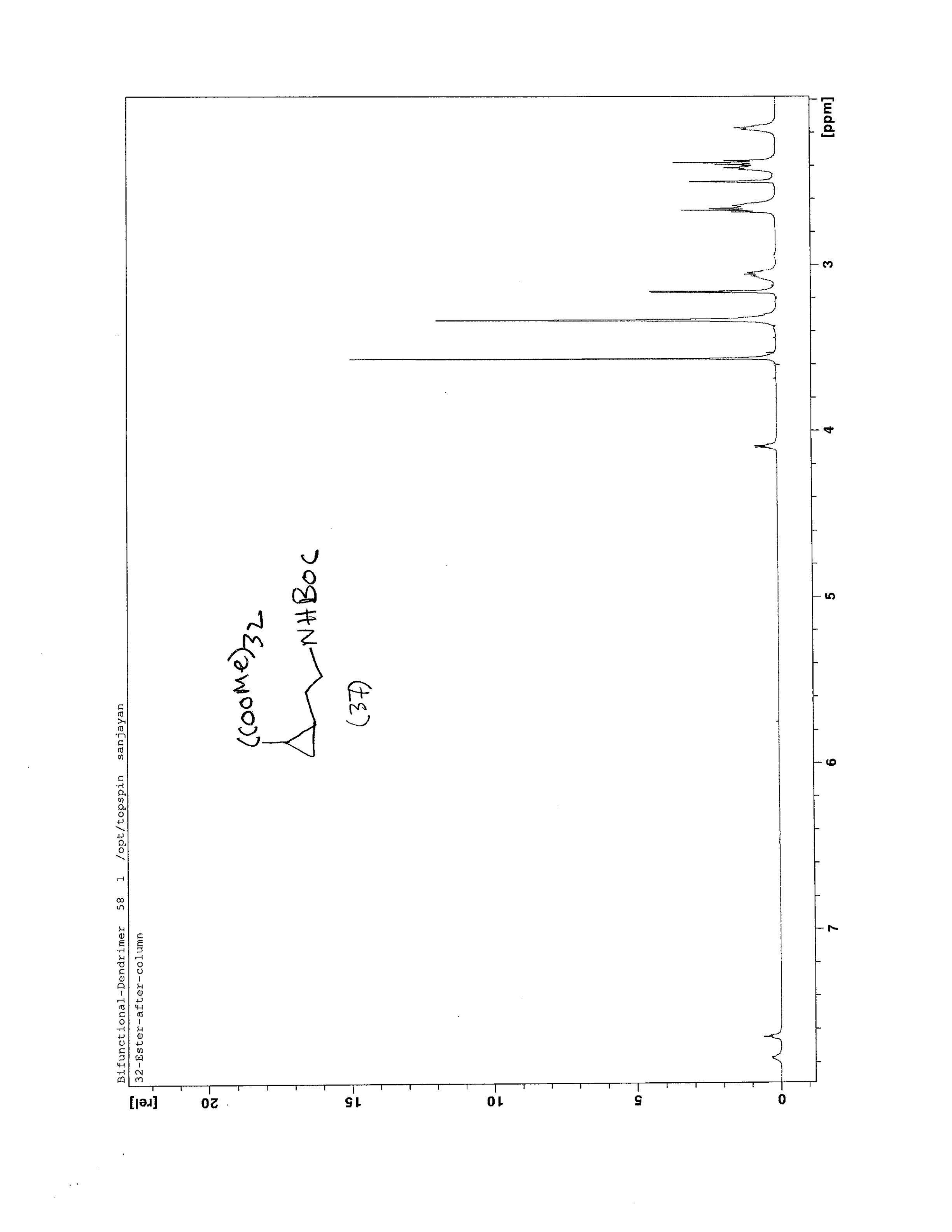
**
