# Supplementary material for: Nucleoside conjugates of quantum dots for characterization of G protein-coupled receptors: strategies for immobilizing A2A adenosine receptor agonists
Source: J Nanobiotechnology. 2010 May 17;8:11. doi: 10.1186/1477-3155-8-11 (PMC2883535; doi:10.1186/1477-3155-8-11)
Supplement: Additional file 6 — Standard fluoresent curves of representative compounds. Compounds 2a and 2b [file 1477-3155-8-11-S6.DOC]

**Additional information for:**

**Nucleoside conjugates of quantum dots for characterization of G protein-coupled receptors: strategies for immobilizing A_2A_ adenosine receptor agonists**

Arijit Das, Gangadhar Sanjayan, Miklos Kecskes, Lena Yoo, Zhan-Guo Gao, and Kenneth A. Jacobson^*^

Additional file 6

Title: Standard fluoresent curves of representative compounds

Description: Compounds **2a** and **2b**

Toluene-soluble QD **2a** in DMSO: Fluorescence as a function of concentration

Water-soluble QD **2b** in DMSO: Fluorescence as a function of concentration
